# Inflammatory bone loss and signaling pathways in periodontitis: mechanistic insights and emerging therapeutic strategies

**DOI:** 10.1038/s41413-025-00478-1

**Published:** 2026-01-03

**Authors:** Rafael Scaf de Molon, Rolando Vernal, Gabriela Ezequiel Oliveira, Joao Paulo Steffens, Edilson Ervolino, Leticia Helena Theodoro, Jeroen J. J. P. van den Beucken, Sotirios Tetradis

**Affiliations:** 1 Department of Diagnostic and Surgery, School of Dentistry, São Paulo State University (UNESP), Araçatuba, São Paulo Brazil; 2 Periodontal Biology Laboratory, Department of Conservative Dentistry, Faculty of Dentistry, Universidad de Chile, Santiago, Chile; 3 Department of Stomatology, Universidade Federal do Paraná, UFPR, Curitiba, Brazil; 4 Department of Basic Sciences, School of Dentistry, São Paulo State University (UNESP), Araçatuba, São Paulo Brazil; 5 Dentistry - Regenerative Biomaterials, Radboudumc, Nijmegen, Gelderland The Netherlands; 6 Division of Diagnostic and Surgical Sciences, UCLA School of Dentistry, Los Angeles, CA USA

**Keywords:** Pathogenesis, Dental diseases

## Abstract

Bone resorption is a vital physiological process that enables skeletal remodeling, maintenance, and adaptation to mechanical forces throughout life. While tightly regulated under the physiological state, its dysregulation contributes to pathological conditions such as osteoporosis, rheumatoid arthritis, and periodontitis. Periodontitis is a highly prevalent chronic inflammatory disease driven by dysbiotic biofilms that disrupt the oral microbiome, leading to the progressive breakdown of the periodontal ligament, cementum, and alveolar bone and ultimately resulting in tooth loss. This review outlines the molecular and cellular mechanisms underlying periodontitis, focusing on osteoclastogenesis, the differentiation and activation of osteoclasts, the primary mediators of bone resorption. Key transcriptional regulators, including NFATc1, c-Fos, and c-Src are discussed alongside major signaling pathways such as Mitogen Activated Protein Kinase (MAPK), Janus Tyrosine Kinase/Signal Transducer and Activator of Transcription (JAK/STAT), Nuclear Factor Kappa B (NF-κB), and Phosphoinositide 3-kinase (PI3K)/Akt, to elucidate their roles in the initiation and progression of periodontal bone loss. These pathways orchestrate the inflammatory response and osteoclast activity, underscoring their relevance in periodontitis and other osteolytic conditions. Hallmark features of periodontitis, including chronic inflammation, immune dysregulation, and tissue destruction are highlighted, with emphasis on current and emerging therapeutic strategies targeting these molecular pathways. Special attention is given to small molecules, biologics, and natural compounds that have the potential to modulate key signaling pathways. Although advances in understanding these mechanisms have identified promising therapeutic targets, translation into effective clinical interventions remains challenging. Continued research into regulating bone-resorptive signaling pathways is essential for developing more effective treatments for periodontitis and related inflammatory bone diseases.

## Introduction

**Fig. 1:**
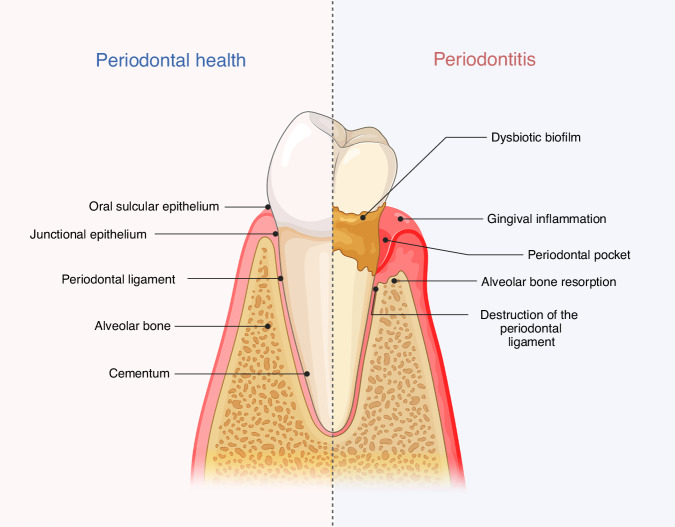
Schematic representation of the periodontium in healthy and diseased conditions. The periodontium’s structure differs notably between healthy and diseased conditions. In a healthy state, the gingiva is securely attached to the tooth surface, and the epithelium, periodontal ligament, cementum, gingiva, and alveolar bone remain structurally sound and undamaged. In contrast, diseased periodontium is marked by periodontal pockets, damage to the periodontal ligament, and decreased bone level due to resorption, which manifests clinically as redness, swelling, and bleeding on probing. Created with BioRender.com (License Number: VX28PGAUQ7)

Bone resorption is a fundamental physiological process that enables remodeling, maintaining, and continuously renewing the skeleton throughout life. However, dysregulation of this process can lead to several pathological conditions, including periodontitis.^1^ Periodontitis, a non-communicable chronic and inflammatory disease linked to a dysbiotic biofilm, affects the teeth-supporting tissues due to progressive destruction of the periodontal ligament, cement, and alveolar bone.^2,3^ (Fig. 1).

**Fig. 2:**
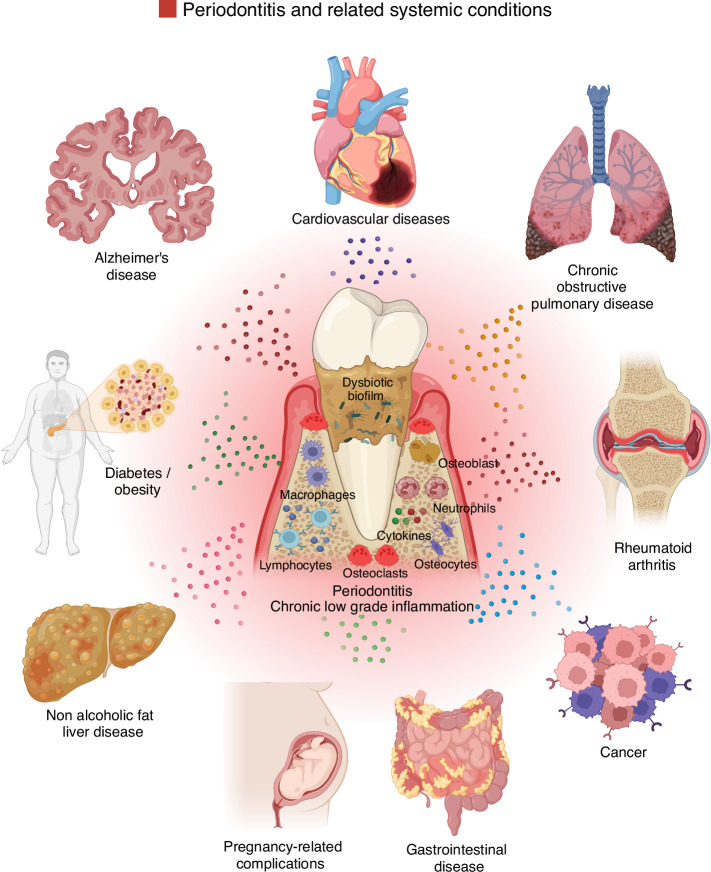
Schematic representation of the influency of periodontitis and chronic low grade inflammation in systemic conditions. The low grade chronic inflammatory condition triggered by microbial byproducts and the host immune response during periodontitis progression driven the dissemination of bacterial components or pro-inflammatory mediators through the circulation. This lead to several non-communicable chronic diseases such as cardiovascular disease, chronic obstructive pulmonary disease, rheumatoid arthritis, cancer, gastrointestinal disease, pregnancy-related complications, non alcoholic fat liver disease, diabetes/obesity, and Alzheimer’s disease. Created with BioRender.com (License Number: FJ28PGB6KS)

This pathological process is intricately linked to the host immune response, which plays a central role in the disease initiation, progression, and exacerbation.^4–7^ Severe periodontitis affects more than 700 million individuals globally, accounting for approximately 11% of the world’s population.^8^ In developed nations, prevalence rates remain high, ranging from 7.2% to 9.8%.^9^ In the United States alone, an estimated 64.7 million adults (46% of the population) are affected by periodontitis, with 8.9% experiencing severe cases.^10^ In Brazil, the severe forms of periodontitis account for almost 20% of the population.^9^ It is recognized as the sixth most prevalent chronic disease worldwide and remains the leading cause of tooth loss among adults.^11,12^ Consequently, periodontitis represents a major public health concern, not only due to its widespread occurrence but also because of its significant impact on oral function and quality of life, including impaired mastication and aesthetic complications.^11^ Furthermore, the financial and societal impact of dental diseases is substantial, with global treatment expenses reaching ~416 billion US dollars annually, accounting for around 5.8% of total healthcare costs.^13^ A significant portion of these expenditures, roughly 75%, is allocated to managing periodontitis. Additionally, periodontitis has been associated with a range of systemic non-communicable chronic diseases, such as rheumatoid arthritis,^14–18^ diabetes mellitus,^19–22^ cardiovascular disease^23–27^, chronic kidney disease,^28^ and others^29–32^ (Fig. 2).

The periodontium is a unique and complex anatomical structure that plays a critical role in supporting the teeth and maintaining oral health. A defining feature of the periodontium is the way in which the teeth penetrate the epithelial barrier, creating a direct and constant interface between the external environment and internal tissues (Fig. 1).^33^ This potentially vulnerable interface is protected by the junctional epithelium (JE), a specialized epithelial tissue that forms a tight seal around the cervical portion of the tooth.^34^ The JE adheres firmly to the tooth surface, whether enamel or cementum, through hemidesmosomes and an internal basal lamina, creating a structurally secure attachment. Functionally, the JE serves as both a biological barrier and an immunological sentinel.^33^ It restricts microbial invasion while allowing selective passage of immune cells, such as neutrophils, macrophages and lymphocytes, thus facilitating immune surveillance and maintaining tissue homeostasis. However, this protective barrier is disrupted during the onset and progression of periodontitis.^34^ Pathogenic microorganisms in the subgingival biofilm stimulate a persistent inflammatory response that weakens the structural integrity of the JE. As a result, the JE begins to migrate apically along the tooth root, and the underlying gingival collagen fibers and connective tissue is progressively degraded mediated by matrix metalloproteinases (MMPs). This degradation marks the transition from gingivitis to periodontitis. As the JE deteriorates, the periodontium becomes increasingly exposed to bacterial pathogens and their virulence factors. Deep periodontal pockets form, providing a niche for anaerobic bacteria and further perpetuating inflammation. Concurrently, the host immune response leads to the resorption of alveolar bone.

Periodontitis begins with bacterial accumulation on the tooth surface, triggering the host’s innate immune response and recruitment of inflammatory cells. Toll-like receptors (TLRs) play a pivotal role in recognizing pathogen-associated molecular patterns (PAMPs), thereby initiating downstream signaling cascades that drive inflammation and tissue destruction in susceptible individuals.^35^ Specifically, TLR2 and TLR4 recognize bacterial components and regulate the expression of key inflammatory mediators such as tumor necrosis factor-alpha (TNF-α), interleukin-1β (IL-1β) and IL-6.^36^ This immune response activates several intracellular signaling pathways, including the Mitogen-Activated Protein Kinase (MAPK), Janus Tyrosine Kinase/Signal Transducer and Activator of Transcription (JAK/STAT), Nuclear Factor Kappa B (NF-κB), and Phosphoinositide 3-kinase (PI3K)/Akt, which together contribute to osteoclastogenesis and periodontal breakdown.^37–39^ A central mechanism in bone remodeling involves the interaction between the receptor activator of nuclear factor kappa B (RANK), its ligand RANKL, and the decoy receptor osteoprotegerin (OPG), which collectively regulates the balance between bone formation and resorption.^40–42^ Despite substantial advances in understanding the pathogenesis of periodontal disease,^35,43,44^ many aspects remain unclear, particularly regarding the complex interplay among environmental, genetic, immunological, and epigenetic factors.

This narrative review provides a comprehensive overview of the signaling pathways implicated in the onset and progression of periodontal disease, focusing on their role in inflammatory bone loss. Additionally, we discuss therapeutic strategies aimed at modulating key molecular pathways, especially those involving NF-κB, JAK/STAT, MAPK, and PI3K/Akt. A mechanistic understanding of these pathological processes provides insight into the rationale behind current pharmacological treatments.

## Pathogenesis of periodontitis

**Fig. 3:**
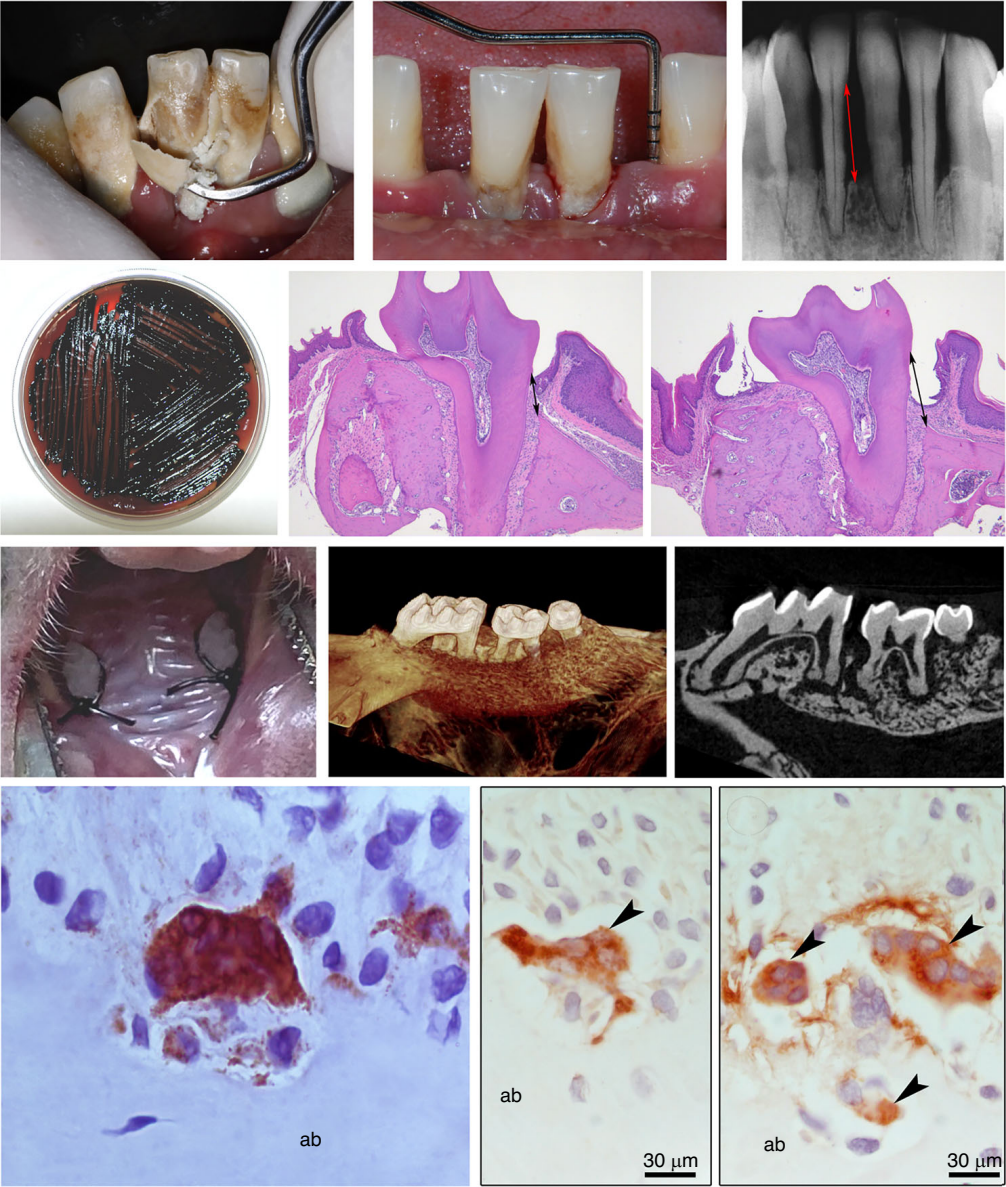
Clinical, radiographic, microbiological, and histological aspects of periodontitis. The upper left image depicts advanced chronic periodontitis, highlighting gingival inflammation and the removal of mineralized bacterial biofilm through supragingival instrumentation. In the upper center, a clinical case of periodontitis is shown, characterized by the formation of periodontal pockets ≥4 mm in depth and alveolar bone loss due to chronic inflammation (measured using a periodontal probe). The upper right figure is a periapical radiograph demonstrating advanced bone loss, with the red line indicating the level of alveolar bone resorption (distance from the cement-enamel junction to the alveolar bone crest). The middle section contains histological images of a mouse maxilla stained with hematoxylin and eosin, comparing healthy and periodontitis-affected tissues. The right middle panel shows a detailed histological view of periodontal inflammation, highlighting infiltrated immune cells and disrupted connective tissue architecture following ligature placement. The center middle panel presents a sagittal view of a healthy tooth. Alveolar bone resorption (marked by an increased black line length) and osteoclastic activity in the periodontitis-affected sample could be observed. The progression of periodontitis results in tissue attachment loss and deepening periodontal pockets, creating an ideal environment for bacterial colonization. Among these, *Porphyromonas gingivalis*, a key periodontitis pathogen, is shown growing on blood agar (center left figure). At the third column, representative images from a mouse model with ligature-induced bone loss in the upper first molars are displayed. The left panel shows the clinical presentation of ligature-induced periodontitis. The middle panel provides a tomographic (micro-CT) reconstruction of the mouse maxilla, highlighting significant alveolar bone loss. The right panel displays a cross-sectional view of the micro-CT image, further illustrating the extent of bone resorption and morphological changes in the periodontal tissues. At the bottom are histological sections stained for TRAP^+^ demonstrating the osteoclasts near the alveolar bone (ab) in a mouse model of induced periodontitis

Periodontitis is a complex, multifactorial disease characterized by a disruption in the balance between host immune responses and environmental triggers.^45^ Rather than resulting from a single infectious agent, periodontitis arises from a chronic inflammatory process within a susceptible host. In healthy conditions, a delicate equilibrium exists between immune surveillance and tolerance, enabling the immune system to prevent inappropriate responses while preserving tissue integrity. This immune balance plays a central role in maintaining periodontal health by preventing exaggerated inflammation.^43,46–50^ When this equilibrium is disturbed, due to genetic predisposition, environmental or local factors, or immune dysregulation, a sustained inflammatory response is triggered. One proposed mechanism involves the presence of a “keystone” pathogen, such as *Porphyromonas gingivalis (P. gingivalis)*, which can perturb host responses even in low abundance.^51^ This leads to an exaggerated and persistent immune activation, ultimately resulting in connective tissue degradation, loss of periodontal attachment, and radiographic evidence of alveolar bone resorption, the hallmark of periodontal disease^50^ (Fig. 3).

**Fig. 4:**
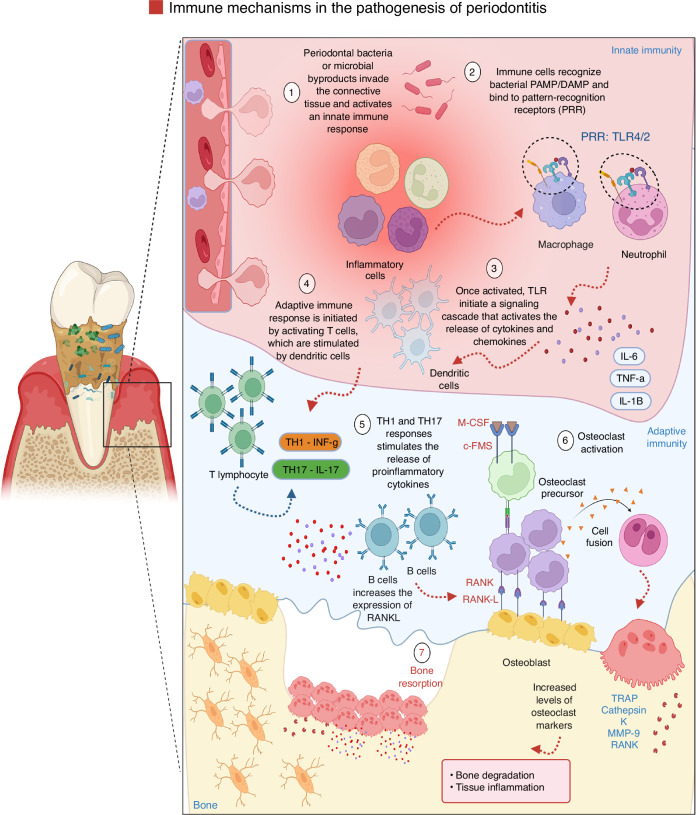
Innate and adaptive immunity drive bone resorption in periodontitis. Innate and adaptive immune responses are central drivers of the inflammatory bone resorption observed in periodontitis. The host response, initiated upon recognition of periodontal pathogenic bacteria such as *Porphyromonas gingivalis*, orchestrates a complex cascade of inflammatory events involving both innate and adaptive immune mechanisms. These immune responses are not only pivotal in pathogen clearance but also contribute to the chronic inflammatory microenvironment that characterizes periodontitis. Stromal cells, including gingival fibroblasts and periodontal ligament cells, interact dynamically with infiltrating immune cells to modulate the local immune milieu. They respond to microbial and inflammatory stimuli by producing chemokines, cytokines, and matrix-degrading enzymes, thereby facilitating immune cell recruitment and perpetuating tissue destruction. Meanwhile, innate immune cells such as neutrophils and macrophages initiate the early inflammatory response, whereas adaptive immune cells, particularly Th17 cells and B cells, amplify inflammation and promote osteoclastogenesis through the secretion of RANKL and other pro-osteoclastogenic factors. Together, stromal and immune cells contribute to the dysregulated immune response and bone remodeling imbalance that underlie the pathogenesis and clinical manifestations of periodontitis. Created with BioRender.com (License Number: ZP28PGCRQ5)

The immunological response involved in periodontal tissue breakdown is dynamic and complex, engaging both the innate and adaptive arms of immunity.^52,53^ The initial response to homeostasis disruption involves rapid recruitment and activation of immune cells in the gingival connective tissue.^54^ The host response is characterized by the activation of the innate immune response, providing a rapid but non-specific line of defense, and the delayed adaptive immune system, which tailors a more targeted and long-lasting response to the infection.^4,55^ The interplay between these immune responses drives the pathogenesis of periodontitis, playing a role in tissue damage and resolution of inflammation (Fig. 4).^7,35^

Host immune activation involves pattern recognition receptors (PRRs), such as TLRs expressed on the membrane of epithelial and immune cells, like macrophages and dendritic cells. These receptors sense danger-associated molecular patterns (DAMPs)^35^ or microbial motifs and initiate signaling cascades that promote the release of pro-inflammatory mediators such as IL-1β, IL-6, and TNF-α.^56^ These cytokines recruit neutrophils to the infection site. Upon sensing infection or tissue injury, neutrophils are rapidly mobilized from the bone marrow into circulation and subsequently recruited to the affected sites. There, they execute a multifaceted antimicrobial arsenal, including phagocytosis, degranulation, the production of reactive oxygen species (ROS), and the release of neutrophil extracellular traps (NETs—extracellular web-like structures composed of decondensed chromatin and antimicrobial proteins), all of which constitute the first line of defense against pathogens.^57,58^ Moreover, neutrophils can contribute to an anti-inflammatory phenotype, characterized by secretion of IL-10 and CCL2, thereby favoring to immune suppression and resolution of inflammation. Conversely, in chronic inflammatory scenarios, some neutrophils exhibit hyperinflammatory profiles, producing pro-inflammatory cytokines such as IL-12 and chemokines like CCL3 and CCL4. These mediators stimulate other immune cells and contribute to the aggravation of tissue breakdown (Fig. 4).^59,60^

Although neutrophils are abundant in the periodontal tissues during active periodontitis and are known to contribute to the inflammatory milieu that supports bone resorption, histopathological studies have consistently shown that these cells are infrequently found in direct proximity to bone surfaces undergoing active osteoclastic resorption.^61,62^ Instead, neutrophils predominantly accumulate within the gingival crevice, junctional epithelium, and connective tissue near the microbial biofilm, where they primarily function to contain bacterial invasion through phagocytosis and the release of antimicrobial peptides and NETs.^63,64^

In human periodontitis, active resorptive sites are typically occupied by osteoclasts and their precursors, which are differentiated under the influence of RANKL and pro-inflammatory cytokines secreted by immune and stromal cells, including T and B lymphocytes, macrophages, and periodontal ligament fibroblasts.^62^ While neutrophils contribute to the inflammatory environment by releasing matrix metalloproteinases (e.g., MMP-8 and MMP-9), IL-1β, TNF-α, and ROS, these factors act predominantly in a paracrine manner, influencing osteoclastogenesis indirectly rather than through direct cell-to-cell contact with osteoclast precursors at the bone surface.^61^

The spatial segregation between neutrophils and resorptive bone sites highlights their primary immunological function in microbial defense rather than direct participation in bone matrix degradation.^65^ However, emerging data suggest that NETs and neutrophil-derived cytokines may sustain chronic inflammation and enhance the expression of RANKL by other immune cells, thereby creating a pro-resorptive environment at a distance.^66,67^ Thus, while neutrophils are not in close anatomical contact with osteoclasts in vivo, their secretory profile may contribute to the amplification of osteoclastogenic signaling cascades that promote alveolar bone loss in periodontitis. Therefore, in the context of osteoclastic bone resorption in periodontitis, neutrophils should be viewed as upstream modulators of inflammation rather than direct effector cells at the bone surface. Recognizing this anatomical and functional distinction is essential to accurately interpreting the contribution of neutrophils to periodontal bone loss.^65,67^

Importantly, neutrophils are now recognized as key players in trained-immunity, a form of innate immune memory characterized by long-lasting functional reprogramming following an initial challenge.^68,69^ Upon exposure to certain microbial components or endogenous stimuli, neutrophils can undergo epigenetic and metabolic reprogramming that enhances their responsiveness to subsequent stimuli, even after the initial trigger has been cleared. This “training” results in augmented effector functions, such as increased production of ROS, enhanced phagocytosis, and elevated pro-inflammatory cytokine release, contributing to heightened pathogen clearance upon reinfection.^68,69^ Recently, Haacke et al.^70^ (2025) uncovers a novel dimension of trained immunity by demonstrating that osteoclastogenesis, the process by which osteoclasts differentiate from myeloid progenitors, is itself subject to innate immune training, thereby exacerbating inflammatory bone loss. Using β-glucan to induce trained immunity (TRIM) in mice, the authors found that this intervention led to a heightened osteoclastogenic predisposition in both bone marrow progenitors and peripheral monocytes. Upon secondary inflammatory challenge (e.g., periodontitis or arthritis), TRIM significantly increased osteoclast precursor populations, elevated expression of osteoclastogenic transcription factors, and enhanced osteoclast formation and activity in affected tissues.^70^ These effects were shown to be dependent on transcriptomic rewiring of monocytes, particularly through MITF signaling, which was essential for the pro-osteoclastogenic phenotype. This study thus identifies “trained osteoclastogenesis” as a maladaptive arm of innate immune memory that may contribute to chronic inflammatory bone loss.^70^

Macrophages play a more critical role in the innate immune response and tissue destruction.^71,72^ Once activated, macrophages release pro-inflammatory cytokines, which amplify the inflammatory response and activate other immune cells. They further secrete catabolic enzymes such as prostaglandins and MMPs, which favor the breakdown of the extracellular matrix, leading to periodontal damage.^71,72^ Additionally, dendritic cells, functioning as antigen-presenting cells, process pathogens and express bacterial antigens, after which they travel to lymph nodes to activate the adaptive immune response. This represents a critical junction between the innate and adaptive immune systems (Fig. 4).^43^

The adaptive immune response is initiated by activating T cells, which are stimulated by antigen-presenting cells, including dendritic cells.^72^ In periodontitis, T helper 1 (Th1), Th2, and Th17 cells are key players in the immune response.^73^ Th1 cells, driven by the transcription factor T-bet and induced by IL-12, produce interferon-gamma (IFN-γ), which activates macrophages and promotes a pro-inflammatory response. Th17 cells, under the control of the transcription factor RORγT, secrete IL-17A, a cytokine that stimulates the production of pro-inflammatory mediators such as IL-6 and TNF-α, as well as MMPs.^74^ These factors contribute to tissue destruction by promoting neutrophil recruitment and activation, and by enhancing osteoclast activity, leading to bone resorption. Th17 cells are critically involved in initiating and sustaining inflammation and bone loss during the early stages of periodontal disease (Fig. 4).^75^ In contrast, Th2 cells, regulated by the transcription factor GATA-3, play a more protective and regulatory role. They secrete anti-inflammatory cytokines, including IL-4, IL-5, IL-9, IL-10, and IL-13, which help modulate the immune response and inhibit osteoclastogenesis, thereby contributing to immune homeostasis and limiting tissue damage.

B cells also play a role in the adaptive immune response and disease progression. They differentiate into plasma cells, which secrete antibodies, primarily immunoglobulin G (IgG), which can specifically bind to periodontal bacteria and facilitate their clearance.^75^ However, in periodontitis, the antibody response may be insufficient to clear the invaders completely, and the persistence of pathogenic insult in the periodontal tissues can result in chronic inflammation and lack of resolution. Recent studies have shown that B-cells can also function as antigen-presenting cells and release inflammatory cytokines, further exacerbating the inflammatory environment.^76,77^ Over time, this leads to the stimulation of osteoclasts, leading to bone resorption and the degradation of the tooth supporting structures (Fig. 4).^35,36^

The resolution of inflammation is a crucial and tightly regulated phase of the immune response that is essential for restoring tissue homeostasis following the cessation of a pro-inflammatory stimulus. Effective resolution depends on a phenotypic switch in immune cells, characterized by a coordinated transition from a pro-inflammatory to a pro-resolving state. This process involves the suppression of pro-inflammatory mediators and the activation of specialized pro-resolving pathways, including the upregulation of anti-inflammatory cytokines such as interleukin-10 and transforming growth factor-beta (TGF-β).^78,79^ These cytokines are key regulators in terminating inflammation by inhibiting further immune cell activation, promoting tissue repair and regeneration, and enhancing the clearance of apoptotic cells through efferocytosis.

Specialized pro-resolving mediators (SPMs), including lipoxins, resolvins, protectins, and maresins, are biosynthesized from omega-3 and omega-6 polyunsaturated fatty acids and serve as key molecular drivers of the resolution phase.^80–83^ These lipid mediators limit neutrophil recruitment, enhance macrophage-mediated efferocytosis, and promote a phenotypic switch in macrophages from a pro-inflammatory (M1) to an anti-inflammatory, tissue-reparative phenotype (M2). Additionally, regulatory T cells (Tregs) expand during this phase and secrete immunosuppressive cytokines that further promote the cessation of inflammation and support mucosal tolerance. In the context of periodontitis, however, this finely tuned resolution program is disrupted.^80–83^ The persistence of microbial biofilms and inadequate plaque control leads to continuous activation of innate immune responses and sustained production of pro-inflammatory cytokines. These mediators perpetuate the inflammatory milieu and impair the production or function of SPMs, thereby blocking the transition to resolution.^84,85^ Neutrophils and macrophages, which under normal circumstances would undergo apoptosis and be efficiently cleared, instead remain activated and accumulate in the periodontal tissues, contributing to extracellular matrix degradation.

Moreover, the failure to effectively clear apoptotic cells (defective efferocytosis) and to reprogram immune cells toward a reparative phenotype exacerbates tissue destruction and prevents healing. Chronic, unresolved inflammation leads to the breakdown of periodontal connective tissue and progressive alveolar bone loss. Thus, the inability to initiate or sustain the resolution phase represents a critical pathogenic mechanism in the chronicity and progression of periodontitis.

**Fig. 5:**
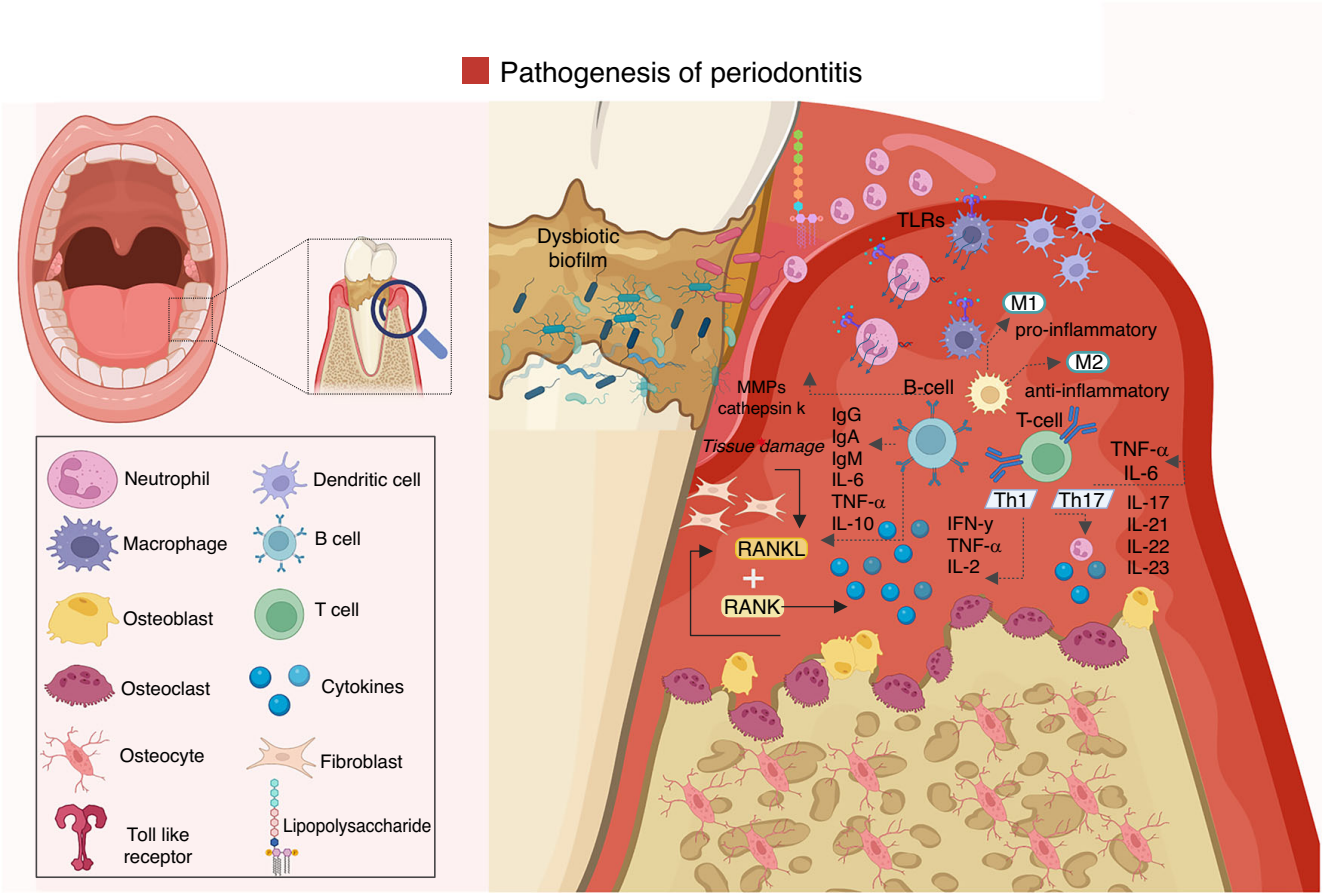
Inflammatory responses related to periodontitis. Periodontitis is characterized by the dysregulated stimulation of the innate host response and is primarily caused by an imbalance in the microbial community due to pathogenic biofilms. This imbalance activates an excessive inflammatory reaction, involving the recruitment of neutrophils, macrophages, and lymphocytes to the periodontal pocket. The condition arises from oral dysbiosis caused by biofilms composed of periodontal pathogens. Virulence factors released by pathogenic bacteria recruit neutrophils to the inflamed gingival tissue. This recruitment triggers excessive pro-inflammatory cytokine production by keratinocytes, fibroblasts, and antigen-presenting cells, leading to a hyperactive innate immune response. Macrophages contribute to this response by increasing the secretion of pro-inflammatory cytokines and MMPs, and activating B cells. The persistent elevation of TNF-α, IL-1β, INF-γ, and MMPs, coupled with the upregulation of TH1 and TH17 responses, promotes the activity of bone-resorbing cells, ultimately leading to osteoclastogenesis and bone destruction. Created with BioRender.com (License Number: GW28PGAZ0X)

In summary, the immune response to periodontal pathogens involves a complex interplay between innate and adaptive mechanisms. While the innate system rapidly responds through PRRs and neutrophil recruitment, the adaptive response, particularly via Th1 and Th17 cells, sustains chronic inflammation. Dendritic cells, macrophages, and B cells contribute to immune activation and tissue destruction. The inability to resolve inflammation and the continued stimulation of osteoclastogenesis underlie the progressive bone loss that defines periodontitis. A detailed understanding of these cellular and molecular events in the pathogenesis of periodontitis is essential for developing effective therapeutic strategies (Fig. 5).^49,86–90^

## Bone remodeling biology: signaling pathways and cellular interactions

Bone homeostasis is governed by two fundamental and continuous processes: remodeling and modeling.^91^ These processes respond to mechanical, hormonal, and nutritional stimuli to ensure bone tissue formation, maintenance, and structural adaptation throughout life.^1,92,93^ Bone remodeling is a dynamic activity that constantly renews bone tissue, preserves its structural integrity, adapts to mechanical load, and maintains mineral balance.^92,94^ In this context, specialized bone cells play key roles: osteoblasts synthesize the bone matrix, while osteoclasts resorb old or compromised bone.^93,95–98^ The balance between synthesis and resorption ensures continuous bone renewal, preventing micro-injury accumulation that could compromise its strength and lead to fractures.^92^

Osteoclasts, the primary bone-resorbing cell, originate from monocyte/macrophage precursors within the hematopoietic lineage in the bone marrow.^99–101^ The migration of osteoclast precursors from the bone marrow and circulation to resorption sites occurs via the collagen network, guided by sphingosine-1-phosphate (S1P) signaling,^102^ produced by sphingosine-1 kinase (SphK) and operating in distinctive S1P receptors.^103,104^ Upon reaching the bone surface, these precursors differentiate into multinucleated, mature osteoclasts with resorptive capacity, a process driven by RANKL and macrophage colony-stimulating factor (M-CSF).^105,106^ These signaling molecules are produced by osteogenic cells, T cells, and vascular endothelial cells near the bone surface. Both soluble (sRANKL) and membrane-bound RANKL (mRANKL) interact with RANK receptors on osteoclast precursors, initiating intracellular signaling cascades mediated by tumor necrosis factor receptor-associated factor (TRAF) proteins, particularly TRAF2, TRAF5, and TRAF6.^99^ This activation subsequently triggers NF-κB and MAPK pathways, leading to the expression of myelocytomatosis oncogene (MYC) and Fos proto-oncogene, AP-1 transcription factor subunit (FOS).^99^ These factors collectively induce the nuclear factor of activated T-cells, cytoplasmic 1 (NFATc1), which plays a crucial role in osteoclast differentiation through the canonical signaling pathway.^107^ As a key transcription factor, NFATc1 facilitates osteoclastogenesis by enhancing the expression of genes associated with bone resorption, including MMP9, cathepsin K, acid phosphatase 5 (Acp5), and tartrate-resistant acid phosphatase (TRAP).^108,109^ Mature osteoclasts are multinucleated, polarized cells that adhere to the bone surface and create a resorption lacuna by secreting lysosomal proteases (Fig. 3), including TRAP, MMPs, and cathepsin K.^110^ Within this acidic microenvironment, bone minerals and organic components are degraded, endocytosed, and subsequently expelled through the secretory domain on the opposite side of the cell.^111^

**Fig. 6:**
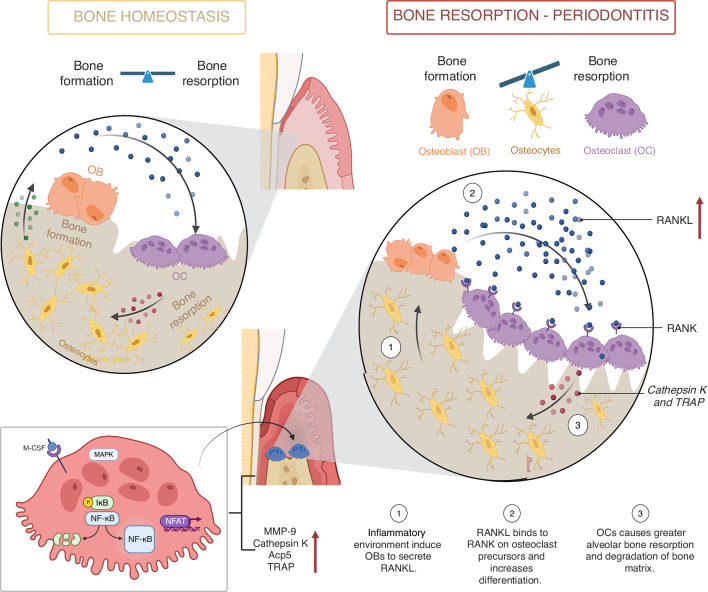
Disruption in the equilibrium concerning osteoclast and osteoblast activity is a key feature of periodontitis. The downregulation of OPG enhances the communication between RANK and its ligand (RANKL), driving osteoclast differentiation and activity. Additionally, various factors, such as IL-1, IL-16, IL-17A, MMPs, and parathyroid hormone (PTH), further promote osteoclast differentiation and bone resorption. Moreover, the signaling pathways involved in bone cell communication are disrupted, leading to impaired bone homeostasis in periodontal disease, as described further below. Created with BioRender.com (License Number: FX28PGB2SL)

The resorption process and the bone remodeling occur in a four-phase cycle: i) *Activation Phase*: Circulating monocyte-macrophages are recruited to the affected site, where they fuse on the bone surface to form multinucleated pre-osteoclasts;^3,92,94,98^ ii) *Resorption Phase:* The resorption process involves osteoclast attachment to the bone matrix and the formation of a ruffled border, which is regulated by integrin-mediated polarization, particularly through α5β1, α2β1, and αvβ3.^100,112–114^ Among them, αvβ3 is the most highly expressed and facilitates osteoclast adhesion to bone matrix proteins, such as osteopontin (OPN), bone sialoprotein, and fibronectin, by recognizing Arg-Gly-Asp (RGD) motifs.^115^ This binding interaction triggers structural adaptations in the osteoclast’s apical membrane and promotes actin ring formation via the phospholipase Cγ2 (Plcγ2), proline-rich tyrosine kinase 2 (Pyk2), and Src signaling pathways, which are critical for bone resorption;^50,92^ iii) *Reversal Phase:* The formed lacunae contain mononuclear cells and osteoblast precursors, which initiate bone formation. Bone resorption activates the release of coupling factors stored within the bone matrix, including TGF-β and insulin-like growth factor-1 (IGF-1). These factors play a crucial role in recruiting osteoblast precursors to the resorption site and facilitating their differentiation, thereby linking bone resorption to bone formation;^92,116^ and iv) *Formation Phase:* Osteoblasts, which secrete osteoid on bone surfaces, comprise approximately 4%–6% of all bone cells and are derived from mesenchymal stem cells,^117^ synthesize the organic matrix for bone mineralization, transporting phosphate and calcium to the mineralization site and degrading inhibitors to allow mineral deposition^50^ (Fig. 6). Some osteoblasts become confined in the matrix and differentiate in osteocytes. Osteocytes maintain bone tissue homeostasis by coordinating the exchange of nutrients and signals through a canalicular network.^1,48,92^ Moreover, osteoblasts and osteocytes can secrete RANKL, OPG, lysophosphatidic acid, and monocyte chemoattractant protein-1 to modulate osteoclast activity.^118^

Osteocytes form a vast three-dimensional network by connecting through their dendritic extensions. This intricate network enables osteocytes to perceive mechanical signals by detecting fluid flow-induced shear stress along their dendrites.^119,120^ In response, they modulate bone’s mechanical properties and interact with osteoblasts and osteoclasts via the RANKL/OPG pathway and the sclerostin/Dickkopf-1/WNT (SOST/Dkk1/WNT) signaling axis.^99^ At the end of the remodeling cycle, ~50%–70% of osteoblasts undergo apoptosis, while the remaining osteoblasts become lining cells or osteocytes.^1,48,92^ Bone lining cells regulate mineral ion transport and can redifferentiate into osteoblasts under certain stimuli, such as mechanical forces or parathyroid hormone.^92^

Recent studies have revealed that mature osteoclasts often undergo fragmentation into smaller, non-resorptive cells known as osteomorphs rather than proceeding to apoptosis.^121,122^ These osteomorphs retain the capacity to migrate across bone surfaces and can rapidly re-fuse into active osteoclasts upon subsequent RANKL stimulation. This recycling mechanism is considered more energy-efficient than de novo osteoclast formation and represents a novel regulatory feature of bone resorption dynamics.^121,122^

## Inflammatory bone loss and osteoclastogenesis

Pathological bone resorption arises when the homeostatic balance of bone formation and resorption is disrupted, leading to excessive osteoclast activity. This can occur through an increase in osteoclast number, function, or both, ultimately resulting in significant degradation of the bone matrix.^94,98^ In periodontitis, chronic inflammation intensifies osteoclastogenesis, leading to progressive destruction of alveolar bone. Central to this process is the RANK/RANKL/OPG axis^48,98,123^ which also plays a pivotal role in the pathogenesis of other skeletal disorders, including osteoporosis, primary or metastatic bone malignancy, autoimmune arthritis, and Paget’s disease.

RANKL, encoded by the *TNFSF11* gene, is a member of the TNF superfamily.^50,94,124–126^ Various cell types, including osteocytes, lymphocytes, osteoblasts, stromal cells, fibroblasts, and periodontal fibroblasts, express RANKL.^50,94,124–126^ In bone, RANKL can be cleaved by MMPs to generate the soluble form sRANKL, primarily secreted by osteoblastic stromal cells.^124–126^ In the periodontium, periodontal ligament cells produce RANKL in response to inflammation, promoting osteoclast differentiation by activating inflammatory signaling cascades and transcriptional regulators.^50,98^ M-CSF, secreted primarily by osteoblasts and fibroblasts, supports the proliferation and survival of mononuclear osteoclast precursors and upregulates the membrane-bound RANK receptor.^48,94,127,128^ RANKL binds to RANK on these precursors, triggering signaling pathways that lead to their differentiation into mature, multinucleated osteoclasts.^124–126^ This process involves activating key transcription factors such as c-Fos, NF-κB, and NFATc1, which are indispensable for osteoclast differentiation and activation.^50^ OPG is a soluble glycoprotein that functions as a decoy receptor for RANKL, competitively inhibiting its binding to RANK and suppressing osteoclastogenesis. The ratio of RANKL to OPG is a critical determinant of bone resorption activity.^48,50,94^ Elevated RANKL levels and reduced OPG expression, commonly observed in periodontal inflammation, create a pro-resorptive environment conducive to alveolar bone loss (Fig. 6).^121^ Previous studies demonstrate that RANKL-deficient (*RANKL*^*−/−*^)^129^ and RANK-deficient (*RANK*^*−/−*^)^130^ mice develop severe osteopetrosis due to impaired osteoclast formation, highlighting the essential role of the RANKL–RANK pathway in bone resorption. Conversely, osteoprotegerin-deficient (*OPG*^*−/−*^) mice exhibit pronounced bone loss, underscoring OPG function as a decoy receptor that inhibits osteoclastogenesis by binding RANKL.^131,132^

The resolution of inflammation plays a pivotal role in suppressing osteoclastogenesis and reestablishing bone homeostasis. This transition involves a coordinated downregulation of pro-resorptive signaling and the promotion of anti-inflammatory and anti-osteoclastogenic pathways. Key cytokines such as IL-10, IL-4, IL-13, and TGF-β act to inhibit osteoclast differentiation by reducing the expression of RANKL and enhancing OPG levels, thereby disrupting the RANK/RANKL axis essential for osteoclast maturation.^133–137^ Tregs further contribute by secreting IL-10 and TGF-β, which not only suppress inflammatory cytokine production but also promote the expansion of M2 macrophages, cells associated with tissue repair and inhibition of osteoclast activity.^133–138^ As levels of pro-inflammatory mediators decline, osteoclast activation is progressively limited, preventing further bone loss. This transition from inflammation to resolution enables osteoblast-mediated bone formation by activating Wnt/β-catenin signaling, bone morphogenetic protein (BMP) pathways, and the release of growth factors such as VEGF and IGF-1. Collectively, these processes restore bone homeostasis, facilitate regeneration, and ensure long-term skeletal stability.

## Osteoclast transcription factors

Transcription factors are critical regulators of gene expression, and their dysregulation can contribute to the development of pathological conditions. In periodontitis, key transcription factors, such as c-Fos, NFATc1, and c-Src play pivotal roles in promoting inflammation and pathological bone resorption by enhancing osteoclastogenesis. Below, we describe the functions and implications of these transcription factors in the context of periodontal disease.

### c-Fos

c-Fos is a component of the activator protein-1 (AP-1) transcription factor complex, which is activated in response to diverse extracellular stimuli, such as mechanical stress and inflammatory cytokines.^139^ It plays a central role in regulating osteoclast differentiation and function.^140^ As a member of the Fos gene family, alongside Fra-1, Fra-2, and FosB, c-Fos forms heterodimers with Jun family proteins (e.g., c-Jun, JunB, JunD) to bind AP-1 DNA motifs and regulate gene expression.^141^

The expression of c-Fos is strongly linked to RANKL signaling. It interacts with other components of the AP-1 complex, including Fosl1, to regulate genes essential for osteoclastogenesis.^142^ c-Fos is a crucial determinant in the lineage commitment of progenitor cells, directing them toward the osteoclast rather than macrophage lineage.^143^ Mice deficient in c-Fos display impaired osteoclast formation and an expansion of bone marrow macrophages, emphasizing its role in skeletal homeostasis and inflammatory bone disease.^144^

High levels of c-Fos are expressed in osteoclast precursors and mature osteoclasts, driving the expression of diverse osteoclast-specific genes.^145,146^ In apical periodontitis^147^ and periodontitis,^148^ c-Fos-mediated signaling enhances osteoclastogenesis and contributes to alveolar bone resorption. Furthermore, mice lacking c-Fos exhibit elevated levels of pro-inflammatory cytokines such as TNF-α, IL-6, and IL-1β, accompanied by reduced expression of IL-10, suggesting that c-Fos modifies the inflammatory response.^149^ Notably, preclinical studies in rat models have demonstrated that pharmacological inhibition of c-Fos attenuates alveolar bone loss, highlighting its therapeutic potential.^150^

### NFATc1

The nuclear factor of activated T-cell cytoplasmic 1 (NFATc1) is a master transcription factor that governs osteoclast differentiation and bone-resorptive activity.^151–154^ NFATc1 is activated downstream of RANKL signaling and promotes the expression of genes encoding key osteoclastic proteins, including cathepsin K, TRAP, and integrin β3.^155^ The initial activation of NFATc1 involves cooperation with NF-κB and NFATc2, with c-Fos playing a supportive role.^156^ The p38 MAPK pathway contributes to the induction of both c-Fos and NFATc1, facilitating sustained NFATc1 expression via an auto-amplification loop.^157^ The crucial role of NFATc1 in osteoclast formation induced by RANKL and TNF is evident from findings showing that both cytokines can still promote osteoclastogenesis in progenitor cells lacking p50 and p52.^158^ NF-κB is essential for heightening the transcription of c-Fos and NFATc1. However, neither c-Fos nor NF-κB appears to be required for initiating the expression of genes critical for progenitor cell differentiation and fusion, supporting the idea that NFATc1 serves as a key regulator of osteoclast formation.^159^ Nevertheless, since osteoclastogenesis in NFATc1-overexpressing progenitors lacking p50/p52 still depended on RANKL or TNF, it appears that NFATc1 alone is insufficient to drive osteoclast differentiation. During periodontitis, increased RANKL levels lead to upregulation of NFATc1, driving osteoclast differentiation and alveolar bone resorption.^155,160^

Animal studies have highlighted the importance of NFATc1 in periodontal bone loss. For example, treatment with a novel benzamide-linked molecule significantly downregulated NFATc1 and other osteoclast-related genes, reducing osteoclastogenesis and protecting against ligature-induced bone loss.^161^ Additionally, mice with myeloid-specific deletion of encoding protein tyrosine phosphatase 1B (Ptpn1) showed diminished osteoclast formation and alveolar bone loss in ligature-induced periodontitis associated with lower NFATc1 expression and reduced RANKL-mediated signaling.^162^ In vitro, osteoclast precursors from Ptpn1-deficient mice exhibited impaired proliferation, decreased expression of osteoclast markers, and suppressed NFATc1 activity.^162^ Moreover, pharmacological inhibition of NFATc1 using CsinCPI-2, a cysteine peptidase inhibitor derived from the orange tree, attenuated alveolar bone destruction in experimental periodontitis, underscoring its therapeutic value.^163^ Notably, combined deficiency of NFATc1 and c-Fos drastically impairs osteoclast fusion and resorptive function, further validating their synergistic role in osteoclastogenesis.^164^

### c-Src

c-Src is a non-receptor tyrosine kinase and chromatin-remodeling factor that modulates gene accessibility and osteoclast function.^151^ Although less extensively studied in periodontitis than c-Fos and NFATc1, c-Src has emerged as a key regulator of bone resorption and inflammatory signaling.^151^ In osteoclasts, c-Src promotes actin ring formation and cytoskeletal organization, essential for bone resorptive activity.^165^ It also modulates transcriptional programs by facilitating the binding of NFATc1 and c-Fos to their target genes. Additionally, c-Src inhibits RUNX Family Transcription Factor 2 (RUNX2), suppressing osteoblast activity and shifting the balance toward bone resorption.^165^ Dysregulation of c-Src may enhance inflammatory responses and exacerbate osteolytic bone loss.^166^

Preclinical studies have begun to unravel the involvement of c-Src in periodontal bone destruction. For instance, lipopolysaccharide (LPS), a component of Gram-negative bacterial cell walls, promotes c-Src expression in bone marrow-derived macrophages via TNF-α signaling. First, LPS induces TNF-α, which upregulates c-Src expression, contributing to osteoclast differentiation and bone resorption.^167^ A recent study by Mao et al. investigated the effects of the immunomodulatory agent C-176, a stimulator of interferon genes (STING) pathway inhibitor, in a mouse model of apical periodontitis. STING-deficient mice exhibited reduced inflammatory infiltration, osteoclast activity, and bone loss compared to wild-type controls.^168^ In vitro, LPS stimulation reduced TRAP^+^ cells and downregulated osteoclast-related genes, including c-Src and Acp5 in macrophage cultures, further implicating c-Src in inflammatory bone loss.^168^

In summary, c-Fos, NFATc1, and c-Src are critical transcription regulators that control osteoclast differentiation, activation, and inflammatory gene expression. Their dysregulation in periodontitis contributes to excessive bone resorption and periodontal tissue destruction. A deeper understanding of their molecular interactions and regulatory networks provides promising avenues for developing targeted therapies to mitigate bone loss and preserve periodontal health.

## Main signaling pathways in periodontitis

The host immune responses are orchestrated by intracellular signaling pathways that convert extracellular cues, including microbial products and cytokines, into changes in gene expression.^39^ Ligand binding to membrane receptors, such as receptor tyrosine kinases and G protein-coupled receptors, initiates signal transduction cascades, i.e., a sequence of events in which a signal is transmitted from the cell surface to the inside of the cell, resulting in a specific cellular response.^169,170^ Dysregulation of signaling cascade, through prolonged kinase activation or phosphatase inhibition, can result in chronic inflammation and tissue damage, as seen in periodontitis.^155,170^ Therefore, a comprehensive understanding of how these pathways regulate immune responses is essential for identifying molecular targets to control periodontal inflammation and bone loss.^39,171,172^ Key signaling pathways in periodontitis include NF-κB, JAK/STAT, MAPK, PI3K/Akt, and Wnt/β-catenin.

### Nuclear factor kappa B (NF-κB) pathway

**Fig. 7:**
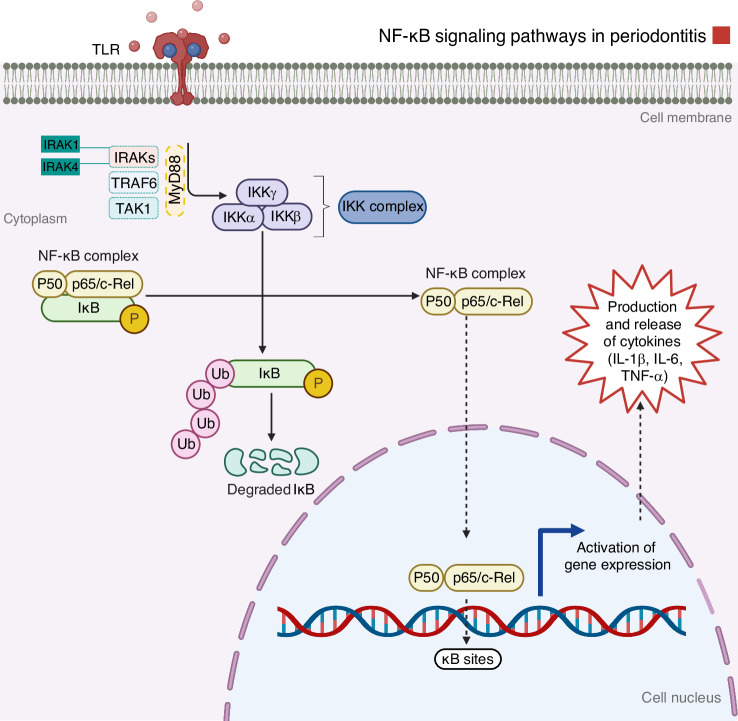
Schematic representation of the NF-κB signaling pathway. The key components of the MAPK pathway involved in signal transduction are highlighted. When a ligand binds, most TLRs attract the connector protein MyD88, activating upstream signaling molecules, including TRAF6, IRAK, and TAK1. These molecules trigger the stimulation of MAP kinase and NF-κB signaling pathways. Additionally, pattern recognition receptors (PRRs) like Nod1 and Nod2 detect peptidoglycan remains from microbial cell walls, which can further enhance TLR-mediated stimulation of these signaling pathways. Once initiated, MAP kinases and NF-κB move into the nucleus, binding to specific promoter regions, including AP-1 and NF-κB, to initiate the transcription of early-response and inflammatory genes. This leads to mRNA production and an increase in cytokine synthesis. Furthermore, p38 MAP kinase plays a crucial role in the regulation of post-transcriptional regulation of pro-inflammatory genes, including IL-6, IL-1, and TNF, by modulating mRNA stability in the cytoplasm. Iwasaki, A. (2025). Adapted from “NF-KB Signaling Pathway.” Retrieved from https://app.biorender.com/biorender-templates. Created with BioRender.com (License Number: RK28PGBXMA)

The NF-κB pathway is a central mediator of inflammation and plays a key role in the immunopathogenesis of periodontitis.^39^ It is activated by microbial components such as LPS and by pro-inflammatory cytokines including TNF-α and IL-1β. NF-κB, a heterodimeric molecule, comprises a family of transcription factors, including c-Rel, RelB, RelA (p65), p50, and p52, which remain inactive in the cytoplasm by binding to inhibitory IκB proteins (Fig. 7).^157^ Upon activation of TLRs, TNF receptors, or IL-1 receptors, adaptor proteins like myeloid differentiation factor 88 (MyD88) and TNF receptor-associated factor (TRAFs) are recruited, triggering the IκB kinase (IKK) complex.^173^ IKK-mediated phosphorylation targets IκB for degradation, allowing NF-κB dimers (e.g., p65:p50) to translocate into the nucleus and induce transcription of genes involved in inflammation, immune cell activation, and survival.^174^

There are two NF-κB activation pathways: (1) The canonical pathway, rapidly triggered by pro-inflammatory cytokines, primarily involves IKKβ and promotes the expression of inflammatory genes via p65:p50.^175^ The p65/p50 heterodimer is translocated to the nucleus, where it binds to specific response elements in the promoters of various osteoclastic genes. (2) The non-canonical pathway, activated by signals such as RANKL and lymphotoxin-β, involves IKKα and activates RelB:p52 dimers, which are important for adaptive immune regulation and lymphoid organogenesis.^176^ Tight regulation of NF-κB is essential to prevent tissue damage. Feedback mechanisms, including de novo IκB synthesis and deubiquitinating enzymes, limit pathway activation.^177^ NF-κB also interacts with other signaling cascades, such as PI3K/Akt and MAPK, to fine-tune the inflammatory response.^178^ Prolonged or dysregulated activation of this pathway contributes to chronic inflammatory and autoimmune conditions.^179^

During periodontitis, NF-κB activation is a key driver of chronic inflammation and bone loss.^39^ Bacterial challenge and immune activation stimulate NF-κB signaling in periodontal tissues, producing IL-6, IL-1β, and TNF-α cytokines.^7^ These cytokines promote the recruitment and activation of macrophages and neutrophils, perpetuating local inflammation.^7^ Importantly, NF-κB regulates osteoclastogenesis by modulating the RANK/RANKL/OPG axis.^180^ Thus, NF-κB signaling contributes directly to alveolar bone loss in periodontitis.^180,181^ Preclinical studies in rat models have shown distinct temporal patterns of NF-κB activation depending on the disease trigger. Ligature-induced periodontitis leads to rapid but transient activation of NF-κB, p38, and ERK, whereas LPS-induced models show delayed but sustained signaling.^172^ Therapeutically, blocking NF-κB activation, either directly or by modulating upstream signals such as TLRs or TRAFs, has been shown to reduce periodontal inflammation and bone resorption.

### Janus kinase/signal transducer and activator of transcription JAK/STAT pathway

**Fig. 8:**
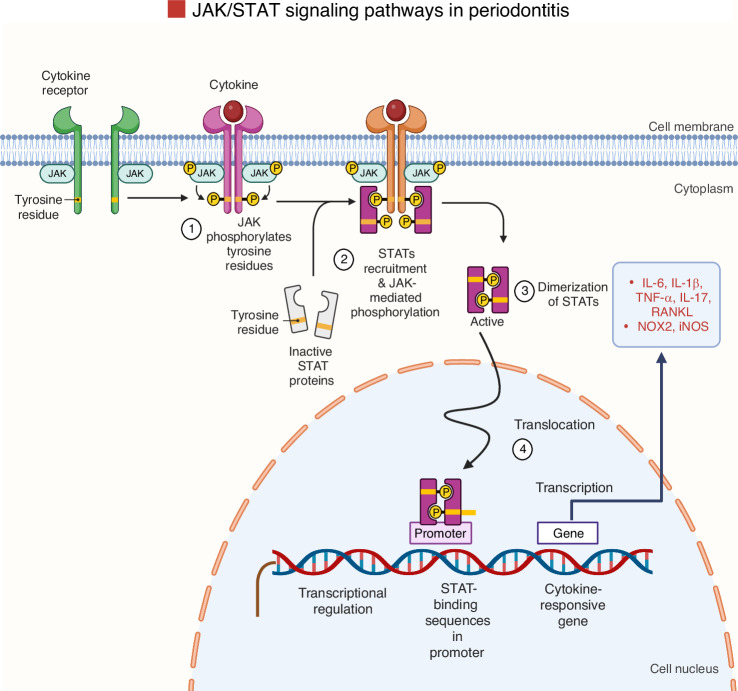
Schematic representation of the JAK/STAT signaling pathway. The main components involved in this pathway are outlined to illustrate the signal transmission process. The initiation of this signaling pathway happens in a series of well-coordinated steps: growth factors and cytokines bind to their corresponding receptors, which leads to receptor dimerization and the recruitment of associated Janus kinases (JAKs). JAK commencement then induces tyrosine phosphorylation of the receptor, generating docking sites for signal transducer and activator of transcription (STAT) proteins. Next, JAKs phosphorylate STATs on tyrosine residues, prompting the STAT proteins to detach from the receptor. These phosphorylated STATs subsequently form either homodimers or heterodimers and move to the nucleus, where they attach to certain DNA structures to regulate the expression of target genes. Iwasaki, A. (2025). Adapted from “Cytokine Signaling through the JAK-STAT Pathway.” Retrieved from https://app.biorender.com/biorender-templates. Created with BioRender.com (License Number: GK28PGBOQ8)

The JAK/STAT pathway is a key mediator of cytokine signaling.^182^ In the context of periodontitis, this pathway is activated by several cytokines and growth factors that bind to membrane receptors, stimulating JAK kinases, which phosphorylate STAT proteins.^182^ Once phosphorylated, STATs dimerize and translocate to the nucleus to regulate gene expression in inflammation and bone remodeling (Fig. 8).^39^

During periodontitis, chronic inflammation driven by microbial infection activates cytokines such as IL-6, IL-1β, TNF-α, and IL-17A, which engage the JAK/STAT pathway and promote the expression of pro-inflammatory mediators and osteoclastogenic factors.^39^ Among the STAT proteins, STAT3 has emerged as particularly relevant in periodontitis. IL-6-induced STAT3 activation promotes M1 macrophage polarization, a pro-inflammatory phenotype, and enhances the expression of osteoclastogenic factors, including RANKL and M-CSF.^39^ Elevated STAT3 phosphorylation has been found in inflamed gingival tissues and is associated with greater disease severity. Experimental inhibition of STAT3 leads to reduced cytokine expression and decreased bone loss in both in vitro and in vivo models.^183^ IL-17A, produced mainly by Th17 cells, also engages the STAT3 pathway and contributes to matrix degradation and osteoclast activity. Collectively, these findings position the IL-6/STAT3 and IL-17A/STAT3 axes as critical drivers of periodontal tissue destruction and potential therapeutic targets. Arce et al.^184^ showed that STAT3 phosphorylation is elevated in gingival tissues during periodontitis, particularly in T cells and keratinocytes. Genetic and pharmacological inhibition of STAT3 reduced alveolar bone loss, reinforcing its pathogenic role.^184^

Suppressors of cytokine signaling (SOCS) proteins, particularly SOCS1 and SOCS3, are endogenous inhibitors of the JAK/STAT pathway.^185^ Studies using LPS-induced and ligature-induced periodontitis models have shown that SOCS3 expression correlates with the severity of inflammation and regulates STAT3 activity.^185^ These findings suggest that modulating SOCS proteins could provide a strategy to control periodontal inflammation and tissue destruction.

### Mitogen-activated protein kinase pathway

**Fig. 9:**
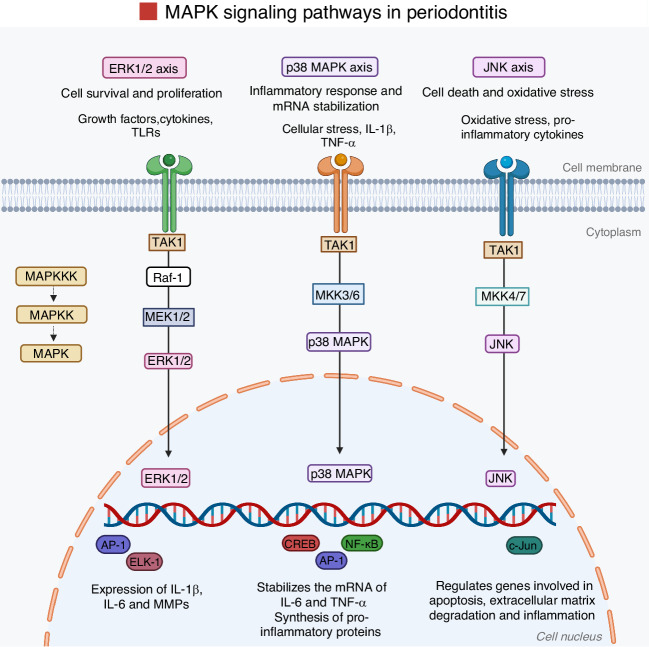
Graphic illustration of the MAPK signaling pathway. The MAPK/ERK pathway components are highlighted to demonstrate signal transmission in periodontal tissues, where PRRs and innate immune signaling pathways play crucial roles. TLRs, such as TLR-2, TLR-4, and TLR-9, are prominently expressed in these tissues. After the binding of a ligand, most TLRs recruit MyD88, an adaptor protein which activates common upstream molecules, such as TAK1, TRAF6, and IRAK, subsequently triggering the MAP kinase and NF-κB pathways. Once activated, these kinases translocate to the nucleus, where they bind to specific promoter motifs, such as NF-κB and AP-1, on target genes, comprising inflammatory genes and early responses, to promote their transcription into mRNA, leading to increased cytokine production. Moreover, p38 MAP kinase contributes to the post-transcriptional regulation of pro-inflammatory genes, such as IL-6, by influencing mRNA constancy into the cytoplasm. Created with BioRender.com (License Number: RI28PGCL8E)

The MAPK pathway is a key signaling cascade that regulates cellular responses to stress, cytokines, and microbial stimuli.^39^ It comprises three main subfamilies: p38 MAPKs, c-Jun N-terminal kinases (JNKs), and extracellular signal-regulated kinases (ERKs). Each of these kinases contributes to the transcriptional regulation of pro-inflammatory genes and the activation of osteoclast-related signaling in periodontal disease.^39^ MAPK signaling is initiated through receptor engagement, such as growth factor or cytokine receptors, which activate a kinase cascade recognized as the Ras-Raf-MEK-ERK pathway, culminating in the phosphorylation of transcription factors and gene expression (Fig. 9).^186^

During periodontitis, pro-inflammatory cytokines such as IL-1β, TNF-α, and IL-17A activate the MAPK pathway, leading to increased expression of MMPs, osteoclastogenic factors, and inflammatory mediators that drive tissue breakdown and bone resorption.^39^ Among the MAPKs, p38 MAPK plays a particularly prominent role.^148^ Inhibition of p38 MAPK suppresses osteoclastogenesis and protects against bone loss in experimental models of periodontitis.^148^ JNK is also involved in periodontitis by regulating the AP-1 transcription factor, composed of c-Jun and c-Fos, essential for osteoclast differentiation.^187^ JNK signaling enhances the expression of inflammatory cytokines and MMPs, and contributes to bone resorption.^179^ Inhibition of JNK promotes bone regeneration and periodontal repair in preclinical models.^188^ Pro-inflammatory stimuli similarly activate ERK signaling and promote osteoclast differentiation and cytokine expression.^189^ Cross-talk between ERK and p38 signaling pathways has been associated with enhances bone resorption in both periodontitis and arthritis.^190,191^

Experimental models support the therapeutic potential of MAPK pathway inhibition.^192^ siRNA targeting MK2, a downstream target of p38, reduces LPS-induced inflammation and bone loss in rats.^193^ Likewise, pharmacological inhibitors such as SD282 (p38 inhibitor) and Bortezomib (a proteasome inhibitor affecting MAPK-related pathways) have been shown to suppress cytokine expression, RANKL upregulation, and bone destruction in periodontitis models.^192^ Bortezomib also reduced the RANKL/OPG ratio and inhibited NF-κB, p38, and ERK activation in LPS-stimulated periodontal ligament cells.^194^ Together, these findings underscore the central role of MAPK signaling, particularly the p38 MAPK and JNK branches, in periodontal inflammation and bone resorption, highlighting these kinases as promising therapeutic targets for periodontitis and related inflammatory bone diseases.

### Phosphoinositide 3-kinase (PI3K)/Akt signaling pathway

**Fig. 10:**
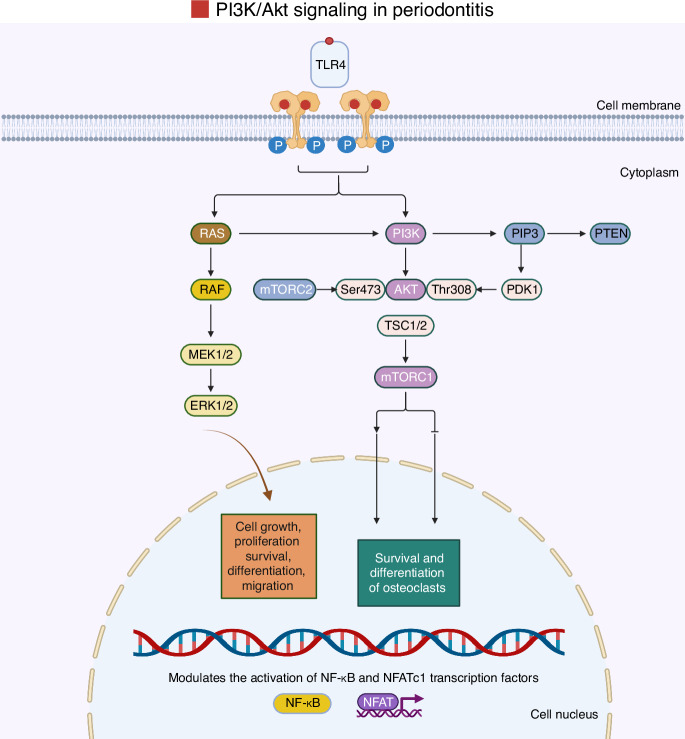
Graphic illustration of the PI3K/Akt signaling pathway. The PI3K-Akt pathway components are highlighted to demonstrate signal transmission in periodontal tissues, where PRRs and innate immune signaling pathways play crucial roles. TLRs, such as TLR-4, are prominently expressed in these tissues. After the binding of a ligand, most TLRs recruit PI3K/Akt, which activates common molecules, such as TSC1/2, subsequently triggering the mTORC1 pathways. Once activated, these kinases translocate to the nucleus, where they bind to specific promoter motifs on target genes, comprising inflammatory genes and early responses, to promote their transcription into mRNA, leading to increased cytokine production. Team, B. (2025). Adapted from “PI3K/Akt, RAS/MAPK, JAK/STAT Signaling”. Retrieved from https://app.biorender.com/biorender-templates. Created with BioRender.com (License Number: PU28PGCWVG)

The PI3K/Akt signaling is involved in a wide range of cellular processes, including cell growth, survival, metabolism, and immune regulation.^195^ Dysregulation of the PI3K/Akt signaling pathway is increasingly recognized as a critical contributor to the pathophysiology of chronic inflammatory and bone-resorptive conditions, including periodontitis.^196^ PI3K activation leads to the production of phosphatidylinositol (3,4,5)-trisphosphate (PIP3), which recruits Akt to the cell membrane, where it becomes phosphorylated and activated. Akt then modulates downstream targets such as mammalian target of rapamycin (mTOR), Forkhead box O1 (FOXO1), GSK3β, and NF-κB (Fig. 10).^196^

Through these downstream effectors, the PI3K/Akt pathway modulates the transcription of genes that govern osteoclast differentiation, matrix metalloproteinase expression, and inflammatory cytokine production.^127,197,198^ In periodontitis, persistent activation of this pathway enhances osteoclastogenesis and promotes the survival of inflammatory cells within periodontal tissues, thereby amplifying tissue destruction and bone loss.^199–202^ Additionally, impaired regulation of PI3K/Akt signaling may disrupt the balance between bone resorption and formation, contributing to the progressive and destructive nature of the disease. Given its central role in coordinating inflammatory and bone remodeling responses, targeting the PI3K/Akt pathway represents a promising therapeutic strategy for mitigating periodontal tissue degradation and preserving alveolar bone integrity.^127,199–202^

Moreover, Akt signaling plays a pivotal role in controlling both the survival and differentiation of osteoclasts.^127^ Within these cells, Akt functions as a key downstream mediator of signaling pathways initiated by three major surface receptors: colony-stimulating factor-1 receptor (c-Fms), αvβ3 integrin, and RANK. Upon stimulation with M-CSF and RANKL, Akt becomes phosphorylated and contributes significantly to osteoclastogenesis, primarily by modulating the activation of transcription factors NF-κB and NFATc1.^197,198^ In a study by Moon et al. (2012), it was demonstrated that Akt facilitates osteoclast differentiation via the GSK3β/NFATc1 axis. Silencing Akt expression using small interfering RNA was shown to effectively block RANKL-induced osteoclast formation.^197^ Furthermore, Akt appears to be particularly important during the early stages of osteoclast precursor development, as its inhibition disrupted osteoclast formation when induced by RANKL at these early differentiation stages, with less pronounced effects at later stages.^197^

### The wingless Int-1 (Wnt)/ β-catenin pathway

The Wnt/β-catenin signaling pathway is fundamental in cell fate determination, tissue homeostasis, and bone remodeling. Upon binding of Wnt ligands to Frizzled receptors, the β-catenin destruction complex is inhibited, allowing β-catenin to accumulate in the cytoplasm and translocate into the nucleus.^203^ There, it activates the transcription of target genes involved in cell proliferation, differentiation, and survival.^203^

Aberrant activation of this pathway, such as through mutations in the adenomatous polyposis coli (APC) gene, can result in excessive β-catenin accumulation and has been implicated in the pathogenesis of several cancers, including colorectal carcinoma.^204^ Although less studied in periodontitis, emerging evidence suggests that dysregulation of Wnt/β-catenin signaling may contribute to the imbalance between bone resorption and formation in periodontal disease.^205–208^

### Pathway interactions

Cell signaling pathways function as integrated networks, with extensive crosstalk allowing fine-tuned regulation of immune responses, tissue remodeling, and cell survival. For instance, the MAPK and NF-κB pathways can act synergistically to amplify inflammation, whereas pathways such as PI3K-Akt modulate cell survival and metabolism.^209^ On the other hand, activation of p38 MAPK initiates the senescence response in both immune and non-immune cells. Recent studies suggest that senescence, particularly stress-induced premature senescence (SIPS), plays a causal role in tooth-supporting bone loss during periodontitis.^210^ Specifically, p38 MAPK: (1) stabilizes and activates p53, a central regulator of the DNA damage response; (2) induces the expression of p16Ink4a and p21Cip1, which inhibit cyclin-CDK complexes and enforce cell cycle arrest; (3) increases mitochondrial reactive oxygen species production and alters antioxidant enzyme expression, thereby disturbing redox homeostasis and promoting mitochondrial dysfunction; and (4) promotes the release of a senescence-associated secretory phenotype (SASP) enriched in RANKL and IL-17A.^211–214^ Collectively, these mechanisms highlight the multifaceted role of p38 MAPK in shaping the senescent phenotype and mediating its pathological impact in periodontitis.^215^ Understanding how these pathways interact is critical for identifying combinatorial therapeutic strategies that modulate inflammatory bone loss in periodontitis.

## Signaling pathways as therapeutic targets

Various host-modulating strategies have been explored to complement periodontal therapy, showing promise in reducing tissue destruction and enhancing clinical outcomes when used alongside non-surgical interventions.^11,49,216,217^ These include anti-inflammatory agents, biochemical mediators, weak organic acids, antibiotics, natural compounds, and bone-protective drugs.^11,49,216,217^

Significant progress has been made in treating osteoclast-driven diseases using agents such as bisphosphonates, denosumab, parathyroid hormone, and estrogen replacement therapy.^218–221^ However, these treatments have notable limitations, including adverse effects, limited suitability for long-term use, and contraindications for oral administration. Additionally, these agents may impair immune function and exacerbate infections in immunocompromised patients or preclinical models.^11,127,217^ As a result, growing attention has turned toward natural compounds for their potential to modulate biological pathways involved in inflammation and bone metabolism.^217^ Active ingredients such as flavonoids, lignans, terpenoids, and limonoids have demonstrated anti-inflammatory, antioxidant, and anti-resorptive properties. These effects are largely mediated by their ability to inhibit RANKL-induced osteoclastogenesis and modulate key signaling pathways, including MAPK, NF-κB, and PI3K/Akt.^11^

**Fig. 11:**
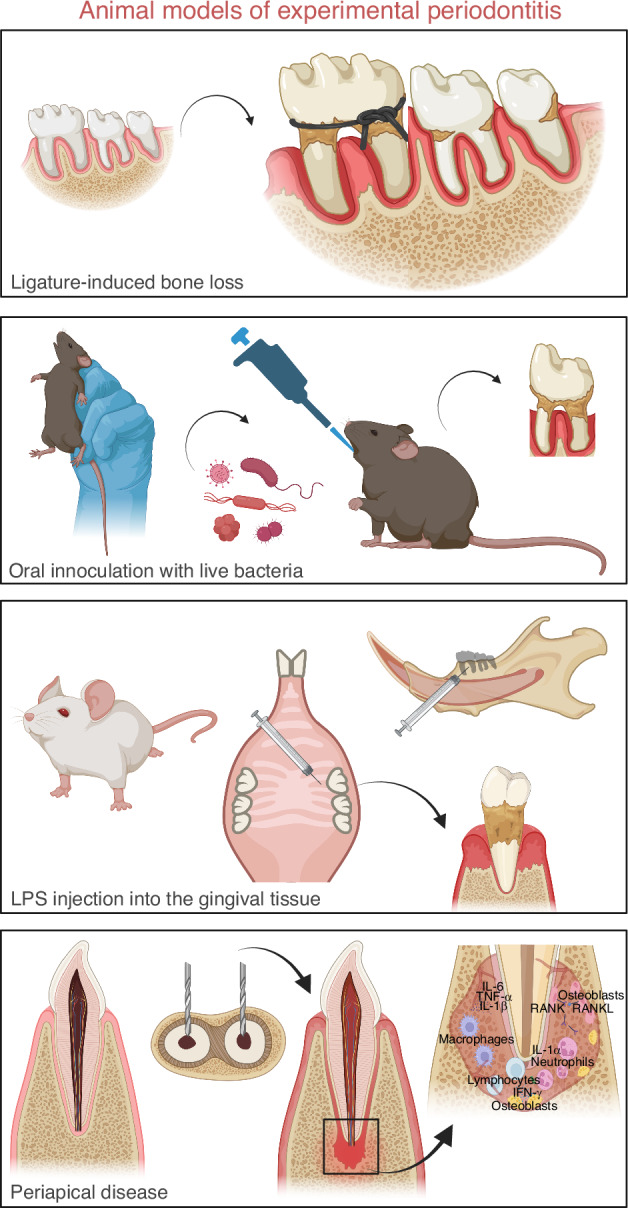
Schematic Representation of Murine Models of Periodontitis Commonly Used to Investigate Therapeutic Strategies Targeting Signaling Pathways. The upper panel illustrates the ligature-induced bone loss model, in which a cotton, silk, or nylon thread is tied around the cervical portion of the teeth. This setup promotes bacterial accumulation, exacerbating the inflammatory process and leading to bone loss. Below, a representative model of oral inoculation with live bacteria is depicted. In this model, live bacteria are cultured under controlled conditions, mixed with carboxymethylcellulose, and applied directly into the animal’s mouth using a pipette. This method facilitates bacterial colonization in the gingival tissue surrounding the teeth, with the severity of tissue inflammation and bone loss being directly proportional to the number of applications and the duration of disease development. The third image in the panel presents a schematic of lipopolysaccharide (LPS) injections, administered directly into the palatal gingiva of the maxilla or mandible using a Hamilton syringe. Repeated LPS application amplifies the inflammatory response and promotes advanced bone resorption. Finally, in the bottom of the panel, the periapical disease model is induced by opening the crown of a tooth, with a round drill, to allow bacteria contamination inside the pulp chamber. The infection leads to the breakdown of alveolar bone surrounding the tooth apex. Created with BioRender.com (License Number: FX28PGAR2G)

In periodontitis, therapeutic efforts increasingly target intracellular signaling cascades that drive inflammation and tissue degradation. Several animal models of induced experimental periodontitis have been widely used in research to unveil the in vivo effects of several different compounds to modulate signaling pathways and decrease inflammation and bone resorption.^222–225^ Some of the most utilized animal models are described in Fig. 11.

Animal models have been instrumental in elucidating mechanisms underlying alveolar bone destruction in periodontitis, emphasizing the pivotal roles of osteoclasts and the innate immune response in health and in genetically modified mouse models.^226,227^ Targeting key intracellular signaling molecules, including NF-κB, c-Fos, and NFATc1, have shown promise as therapeutic strategies in several animal studies.^49,216,217,226^ A review article made by Koide et al.^226^ elegantly examines how osteoblasts and bone marrow stromal cells control osteoclast differentiation and activity, as well as the mechanisms by which bacterial pathogens contribute to bone destruction in periodontitis. Potential treatment strategies to mitigate alveolar bone loss in periodontal disease, including inhibition of RANKL-RANK interaction, signaling molecules (NF-κB, c-Fos, and NFATc1) in osteoclast precursors, and inhibition of osteoclast function, were discussed and summarized in the next sections of this manuscript.

### Therapeutic strategies targeting the NF-κB signaling pathway

**Fig. 12:**
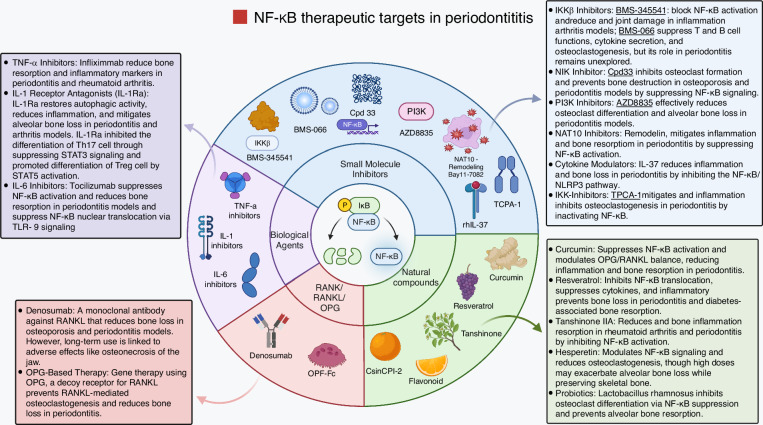
Therapeutic Strategies Targeting the NF-κB Signaling Pathway. Various strategies have been proposed to modulate the alveolar bone resorption, including NF-κB signaling and its downstream effects, aiming to reduce inflammation and prevent excessive bone resorption, including small molecule inhibitors, natural compounds, biological agents, and strategies targeting RANK/RANKL/OPG. This figure illustrates and highlights all the beneficial effects of the aforementioned inhibitors on periodontitis. Created with BioRender.com (License Number: AL28PGAMUR)

Given its central role in inflammatory bone loss, the NF-κB pathway represents a promising therapeutic target in periodontitis and other osteolytic diseases such as rheumatoid arthritis and osteoporosis. Several natural compounds, most notably curcumin and resveratrol, as well as small-molecule inhibitors targeting IκB kinases (IKKs) or NF-κB itself, have shown potential in reducing inflammation and bone resorption in rodent models of experimental periodontitis.^228–230^ Biological agents that neutralize TNF-α or IL-1β, key cytokines downstream of NF-κB activation, have been successfully used in treating systemic inflammatory disorders and may hold similar therapeutic value in periodontal disease and alveolar bone preservation.^231,232^ Overall, a range of therapeutic approaches has been proposed to modulate NF-κB signaling or its downstream mediators to suppress chronic inflammation and mitigate excessive osteoclastic activity in periodontitis (Fig. 12).

## Small molecule inhibitors

Small molecule inhibitors targeting NF-κB signaling have shown promise in managing inflammatory bone conditions such as periodontitis and rheumatoid arthritis.^233–235^ These compounds primarily act by inhibiting key kinases involved in NF-κB activation, including IKKβ and NIK.

- BMS-345541, a selective IKKβ inhibitor, significantly reduces disease incidence and joint inflammation in collagen-induced arthritis models.^233^ Similarly, BMS-066, a newer IKKβ inhibitor, exhibits immunosuppressive effects at low doses by inhibiting Th17 activity, cytokine secretion, and osteoclastogenesis despite limited TNF-α inhibition.^234^ Although not yet studied in periodontitis, these findings support IKKβ as a viable target for inflammatory bone loss.
- Cpd33, an NF-κB-inducing kinase (NIK) inhibitor, suppresses RANKL-induced osteoclastogenesis and reduced bone resorption in both osteoporotic and periodontitis animal models, highlighting its role in modulating the non-canonical NF-κB pathway.^236^ Notably, local administration of Cpd33 in ligature-induced periodontitis mice reduces osteoclast numbers and the expression of inflammatory cytokines, including RANKL, in periodontal tissues, supporting its potential as a therapeutic agent for periodontitis.^236^
- AZD8835, a dual PI3K inhibitor, downregulates NF-κB, ERK, and AKT signaling pathways in vitro and attenuates alveolar bone loss in vivo.^237^ Using a rat model of periodontitis, AZD8835 effectively inhibited osteoclast differentiation and bone resorption, while suppressing the expression of key osteoclast markers, including TRAP and Cathepsin K.^237^
- NF-κB essential modulator (NEMO) binding domain (NBD), a cell-penetrating peptide inhibitor targeting the IκB kinase complex, effectively blocked RANKL-induced NF-κB activation and suppressed osteoclast formation in both cellular and animal models.^238^ These results suggest that specifically targeting NF-κB signaling could serve as a promising treatment strategy for chronic inflammatory conditions associated with excessive bone loss.^238^
- NAT10, an acetyltransferase involved in mRNA modification, has been shown to promotes inflammation through the NOX2–ROS–NF-κB axis.^239^ In an LPS-induced periodontitis mouse model, treatment with Remodelin, a selective NAT10 inhibitor, reduces macrophage infiltration, suppresses pro-inflammatory cytokine expression, and attenuates alveolar bone resorption.^239^
- Deferoxamine (DFO), a hypoxia mimetic, inhibits NF-κB activation and osteoclastogenesis in vitro and in vivo, enhancing HIF-1α expression.^240^ In a rat periodontitis model, DFO reduces inflammation and osteoclast formation by impairing F-actin ring assembly, downregulating osteoclast-specific markers, and attenuating alveolar bone loss.^240^
- Pirfenidone (PFD) significantly reduced alveolar bone loss and suppresses RANKL-induced TRAP^+^ osteoclast differentiation in a ligature-induced periodontitis mouse model.^150^ Additionally, PFD inhibits the production of pro-inflammatory cytokines, including IL-1β, IL-6, and TNF-α, by blocking NF-κB signaling.^150^
- IL-35, an anti-inflammatory cytokine, inhibits alveolar bone loss and modulates the immune response in a ligature-induced periodontitis mouse model.^241^ It reduces osteoclast number and RANKL expression while increasing OPG levels in periodontal tissues. IL-35 also suppressed Th17 cells and associated cytokines (IL-6, IL-17A, IL-23) while enhancing Treg cells and the expression of IL-10, TGF-β1, and IL-35Ebi3. Notably, it decreases RANKL Th17 cells and the RANKL/OPG ratio, supporting its dual role in immune regulation and bone preservation.
- IL-37, an anti-inflammatory cytokine, reduces alveolar bone loss, osteoclast numbers, and pro-inflammatory cytokine expression in a periodontitis model. Specifically, treatment with recombinant human IL-37 (rhIL-37) significantly decreases IL-1β, IL-6, and TNF-α levels, while increasing IL-10 expression in periodontal tissues.^242^ These effects are mediated by suppressing the NF-κB/NLRP3 signaling axis.^242^
- TPCA-1, an IKK inhibitor, exhibits dual effects: while promoting osteoclastogenesis in vitro under M-CSF and RANKL stimulation, it suppresses LPS-induced cytokine production and NF-κB activation, ultimately reducing alveolar bone loss in vivo.^243^
- A novel NIK inhibitor, 4-(3-((7H-pyrrolo[2,3-d]pyrimidin-4-yl)amino)-4-morpholinophenyl)-2-(thiazol-2-yl)-but-3-yn-2-ol, reduces osteoclastogenesis and inflammatory gene expression, and reverses alveolar bone loss in a chronic periodontitis model through inhibition of the non-canonical NF-κB pathway.^244^

These findings collectively support the therapeutic potential of small molecule inhibitors targeting canonical and non-canonical NF-κB signaling in periodontitis, offering novel avenues for inflammation and bone loss control.

## Natural compounds

Several natural compounds have demonstrated the ability to inhibit NF-κB signaling, suppress inflammation, and prevent alveolar bone loss during periodontitis.

- Curcumin, a polyphenol derived from *Curcuma longa*, is a well-characterized NF-κB inhibitor that acts by blocking the phosphorylation of IκB proteins.^245^ In vitro and in vivo studies have shown that curcumin reduces pro-inflammatory cytokine expression, improves the OPG/RANKL ratio, and suppresses NF-κB activation.^230^ A chemically modified form, CMC 2.24, exhibits enhanced therapeutic efficacy in LPS-induced and diabetes-associated periodontitis rat models, significantly reducing inflammatory mediators, MMPs, and bone resorption by inhibiting p38 MAPK and NF-κB pathways.^230^ Notably, when CMC 2.2 is administered orally, it retains efficacy without altering systemic metabolic parameters such as blood glucose or body weight. These findings support the potential of curcumin and its derivatives as adjunctive therapies for periodontitis.
- Resveratrol, a polyphenol found in red wine, has demonstrated protective effects in periodontitis by inhibiting NF-κB signaling and reducing pro-inflammatory cytokine expression.^246^ In experimental models, it suppresses inflammation, osteoclast activity, and NLRP3 inflammasome activation.^246^ In diabetic mice with ligature-induced periodontitis, resveratrol improves glycemic control and attenuates bone loss, partly through TLR4 inhibition and downstream suppression of STAT3, p38 MAPK, and NF-κB pathways.^228^ Notably, it reduces the production of several cytokines, including TNF-α, IL-1β, IL-8, and IL-6.^228^ Encapsulation of resveratrol in nanoparticles further enhances its immunomodulatory effects in models of periodontitis and diabetes.^247,248^ A chitosan-based thermosensitive injectable self-assembled hydrogel (TISH) loaded with granulocyte-macrophage colony-stimulating factor (GM-CSF) and resveratrol (TISH(GR)) to modulate immune responses and promote healing in periodontitis complicated by metabolic syndrome was evaluated.^249^ In vitro experiments revealed that TISH(GR) suppressed inflammatory signaling pathways (MAPKs and NF-κB) in dendritic cells (DCs) and promoted a tolerogenic phenotype, reducing pro-inflammatory cytokines (TNF-α, IL-6) while elevating anti-inflammatory IL-10 cytokine. In a high-fat diet (HFD)-induced periodontitis rat model, adjuvant therapy with TISH(GR) and scaling and root planing (SRP) significantly reduced inflammation and tissue destruction compared to SRP alone, as evidenced by decreased Th17 cell infiltration and enhanced periodontal healing. These findings highlight the potential of TISH(GR) as an immunomodulatory therapy to improve outcomes in periodontitis patients with metabolic comorbidities.^249^
- Tanshinone IIA, a lipophilic diterpenoid quinone derived from *Salvia miltiorrhiza*, a traditional Chinese medicinal herb, exerts anti-inflammatory and anti-resorptive effects by inhibiting Cathepsin K activity and suppressing NF-κB signaling.^247,248,250^ In experimental periodontitis, tanshinone IIA and its sulfonate derivative significantly reduce inflammatory cell infiltration, osteoclast numbers, and the expression of IL-1β, IL-17A, and MMP-13, while improving bone density and collagen fiber integrity.^251^
- Boldine, an aporphine alkaloid from the Chilean tree *Peumus boldus*, exerts anti-resorptive and immunomodulatory effects in ligature-induced periodontitis.^252^ It significantly reduces alveolar bone loss and osteoclast numbers in a dose-dependent manner, downregulates RANKL, and increases OPG levels in periodontal tissues. Boldine also modulates the local immune profile by suppressing Th17 markers (IL-6, IL-17A, IL-23, RORγt) and enhancing Treg-associated cytokines and transcription factors (IL-10, IL-35, TGF-β1, Foxp3), leading to a lower RANKL/OPG ratio and restoration of Th17/Treg balance.
- Hesperetin, a flavonoid found in citrus fruits, inhibits RANKL-induced osteoclastogenesis and bone loss during osteoporosis by downregulating c-Fos, NFATc1, and other osteoclast markers via inhibition of MAPK and NF-κB pathways and activation of Nrf2/HO-1 signaling.^253^ Interestingly, while it prevents femoral bone loss under physiological conditions, high-dose oral administration aggravates alveolar bone loss and osteoclastogenesis in periodontitis, suggesting tissue-specific effects.^254^
- Lactobacillus rhamnosus extract, a probiotic-derived compound, inhibits osteoclast differentiation in vitro by suppressing the NF-κB/c-Fos/NFATc1 axis via TLR-2 signaling.^255^ In vivo, it reduces alveolar bone resorption in a murine periodontitis model, supporting the therapeutic potential of probiotic-based interventions.^255^

## Biologic agents

Biologic therapies targeting cytokines downstream of NF-κB activation have shown efficacy in treating inflammatory bone diseases, including periodontitis.

- TNF-α inhibitors, such as adalimumab and infliximab, are widely used in inflammatory conditions. Infliximab reduces bone resorption and improves bone mineral content in rheumatoid arthritis by inhibiting TNF-α-induced NF-κB activation.^256^ In a ligature-induced periodontitis rat model, infliximab (5 mg/kg) significantly reduces alveolar bone loss, osteoclast numbers, and pro-inflammatory markers (MPO, TNF-α, IL-1β), while improving collagen fiber organization and suppressing RANK, RANKL, MMP-1, and MMP-8 expression.^257^
- Anakinra, an IL-1 receptor antagonist (IL-1Ra), inhibits NF-κB signaling and has demonstrate protective effects in inflammatory diseases.^258^ In osteoarthritis models, IL-1Ra restored autophagy and reduced extracellular matrix degradation by modulating Akt/mTOR/NF-κB and ULK1 signaling.^259^ In collagen-induced arthritis, IL-1Ra reduces inflammation, bone damage, and Th17 responses while enhancing Treg cell differentiation through STAT3/STAT5 modulation.^260^ In periodontitis-affected rats, IL-1Ra delivered via a thermosensitive hydrogel reduces TNF-α, IL-6, and IL-1β expression and alveolar bone loss in diabetic rats, highlighting its therapeutic potential.^261^
- Tocilizumab, a monoclonal antibody targeting the IL-6 receptor, blocks IL-6-mediated NF-κB activation.^262^ In ligature-induced periodontitis, tocilizumab reduces alveolar bone resorption, inflammatory infiltrates, and the expression of RANKL and Th17-related cytokines, suggesting its utility in controlling IL-6-driven periodontal inflammation.^263^

## Targeting the RANK/RANKL/OPG axis

The RANK-RANKL-OPG signaling axis is central to osteoclast differentiation and bone resorption. Dysregulation of this pathway, particularly an increased RANKL/OPG ratio, is a hallmark of periodontitis and drives pathological bone loss. Targeting this axis has emerged as a promising therapeutic strategy for osteolytic diseases, including periodontitis.

- A previous study showed that *OPG*^*−/−*^ mice exhibit more severe alveolar bone resorption, particularly in cortical areas, compared to RANKL-overexpressing transgenic (Tg) mice, despite having higher circulating RANKL levels.^264^ This finding suggests that OPG deficiency exerts a more pronounced effect on bone resorption than RANKL overexpression alone. Microcomputed tomography and histological analysis revealed increased osteoclast activity and cortical porosity in *OPG*^*−/−*^ mice, while RANKL-Tg mice showed milder trabecular bone loss.^264^ Immunohistochemical staining identified osteocytes as a major source of OPG in cortical bone, indicating a protective role against osteoclast-mediated degradation. Additionally, systemic bone resorption markers such as serum TRAP5b activity and the RANKL/OPG ratio correlated with the severity of alveolar bone loss.^264^ Treatment of *OPG*^*−/−*^ mice with either anti-RANKL antibodies or risedronate significantly reduced bone loss and osteoclast numbers, with the anti-RANKL antibody showing particularly robust effects. These findings position *OPG*^*−/−*^ mice as a valuable model for screening anti-resorptive therapies, offering a reliable system to mimic accelerated bone destruction without the need for bacterial induction.^226,264^
- Denosumab, a monoclonal antibody against RANKL, has demonstrated potent anti-resorptive effects and is widely used in osteoporosis and bone malignancies. In postmenopausal women, denosumab significantly reduced bone loss.^265^ In experimental periodontitis models, RANKL-Fc and OPG-Fc therapies also decreased osteoclast activity and alveolar bone resorption induced by *P. gingivalis* infection.^266^ However, denosumab use is associated with adverse effects, including osteonecrosis of the jaw and atypical femoral fractures, which limit its applicability in periodontitis.^267–270^
- OPG acts as a decoy receptor for RANKL, preventing its interaction with RANK and thereby inhibiting osteoclastogenesis. Gene therapy with OPG reduces alveolar bone loss in mice by blocking RANKL–RANK binding.^271^ OPG-based approaches have shown promise in mitigating bone resorption in inflammatory diseases, including periodontitis.^272^ However, similar concerns about osteonecrosis of the jaw will likely limit OPG-centered approaches for periodontitis management.

In summary, therapeutic strategies targeting the RANK-RANKL-OPG pathway effectively control bone destruction in periodontitis. While biologics such as denosumab show strong efficacy, safety concerns highlight the need for more targeted and context-specific approaches.

### Therapeutic strategies targeting the JAK/STAT pathway

**Fig. 13:**
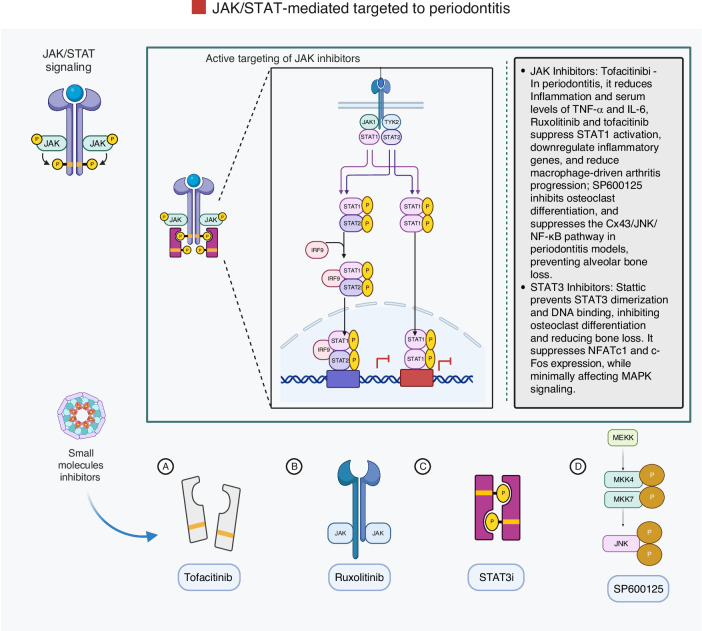
Therapeutic Strategies Targeting the JAK/STAT Signaling Pathway. Various strategies have been proposed to modulate bone resorption, including JAK/STAT signaling and its downstream effects, aiming to reduce inflammation and prevent excessive bone resorption, including small molecule inhibitors and STAT inhibitors. This figure illustrates and highlights all the ameliorative effects of the aforementioned inhibitors on inflammatory bone loss. Created with BioRender.com (License Number: JH28PGAI6J)

Given the central role of JAK/STAT signaling in mediating inflammation and bone resorption, several therapeutic approaches have been developed to modulate this pathway in periodontitis (Fig. 13).

- JAK inhibitors (JAKi) are small molecules that block JAK activation and subsequent STAT phosphorylation. Tofacitinib, a pan-JAK inhibitor, has demonstrated efficacy in the treatment of rheumatoid arthritis and inflammatory bowel disease.^273^ In periodontitis patients, tofacitinib reduces serum TNF-α and IL-6 levels and improves periodontal inflammation after three months despite unchanged bacterial plaque levels.^274^ In vitro and in vivo studies further support its immunomodulatory activity via inhibiting STAT1 and downregulating inflammatory genes in macrophages.^275^ However, JAKi may also enhance osteoclastogenesis through upregulation of c-Jun and NFATc1, warranting careful evaluation of their long-term effects on bone metabolism.^275^
- STAT3 inhibitors also offer therapeutic promise. Stattic, a small-molecule inhibitor of STAT3 dimerization and DNA binding, suppresses RANKL-induced osteoclastogenesis by downregulating NFATc1 and c-Fos expression and attenuates bone loss in an ovariectomy model.^276^ Stattic also inhibited STAT3 and NF-κB signaling, with minimal impact on MAPK pathways, supporting its potential role in managing inflammatory bone diseases; however, further studies in periodontitis are needed.^276^

Altogether, JAK/STAT-targeting agents, including JAK inhibitors, STAT3 blockers, and cytokine modulators, show promise for controlling periodontal inflammation and bone loss. Nevertheless, their clinical application requires further investigation to ensure safety and efficacy, particularly regarding immune suppression and long-term bone health.

### Therapeutic strategies targeting the MAPK pathway

**Fig. 14:**
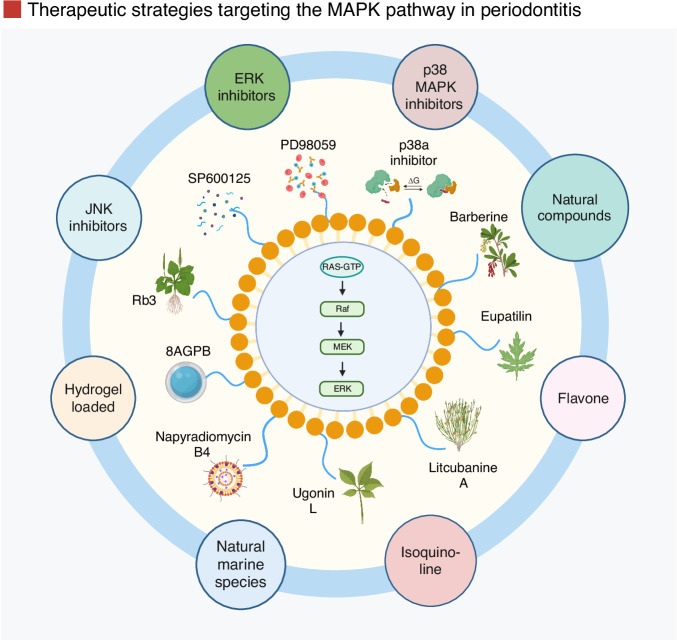
Therapeutic Strategies Targeting the MAPK Signaling Pathway. Various strategies have been proposed to modulate bone resorption, including MAPK signaling and its downstream effects, aiming to reduce inflammation and prevent excessive bone resorption, including ERK, JNK and p38 inhibitors, natural compounds, and hydrogel loaded. This figure illustrates and highlights all the beneficial effects of the aforementioned inhibitors on periodontitis. Created with BioRender.com (License Number: DV28PGACMW)

Given the central role of MAPK signaling in inflammation and osteoclastogenesis in periodontitis, several therapeutic strategies have been explored (Fig. 14).

- Among them, p38 MAPK inhibitors are particularly promising, as they block the expression of MMPs and pro-inflammatory cytokines, thereby reducing osteoclast differentiation.^38^ In response to bacterial stimuli such as LPS, p38 MAPK activation increases cytokine expression and bone resorption.^38^ In a periodontitis model in mice, oral administration of a p38α inhibitor reduces cytokine levels and alveolar bone loss.^277^ MKP-1 overexpression further dampens p38 activity, highlighting its regulatory role in periodontitis.^277^
- Berberine, a natural alkaloid, inhibits p38 MAPK and NF-κB signaling, reducing inflammation and bone resorption in ligature-induced periodontitis.^278^ Delivery via thermo-sensitive hydrogels enhances its anti-inflammatory and osteogenic effects through PI3K/AKT modulation.^279^
- Eupatilin, a flavone from *Artemisia argyi*, blocks RANKL-induced differentiation of Raw264.7 cells and bone marrow-derived macrophages (BMM) into mature osteoclasts.^280^ In vivo, Eupatilin also suppresses RANKL-induced osteoclastogenesis and MAPK/NF-κB signaling, reducing bone loss.^280^
- Litcubanine A (LA), an isoquinoline alkaloid from *Litsea cubeba*, exhibits anti-inflammatory effects by inhibiting macrophage chemotaxis and osteoclast differentiation. In periodontitis models, LA reduces bone loss by downregulating chemokine signaling and suppressing MAPK pathway activation.^281^ Similarly, Lycii Radicis Cortex (LRC), derived from *Lycium chinense Mill*, decreases bacterial load, oxidative stress, and inflammation while preserving connective tissue and alveolar bone via MAPK/NF-κB modulation and RANKL/OPG regulation.^282^
- Napyradiomycin B4 (NB4), a marine-derived compound, inhibited RANKL-induced osteoclastogenesis by suppressing NFATc1 and MEK-ERK signaling, reducing alveolar bone loss in periodontitis mice.^283^ Likewise, Ugonin L, from Helminthostachys zeylanica, suppresses osteoclast differentiation, promotes apoptosis of mature osteoclasts, and inhibits ERK, p38, JNK, and NF-κB activation.^284^
- An immunomodulatory hydrogel (8AGPB) releasing 8-aminoguanosine (8AG) targeted second messengers in macrophages, modulating MAPK and NF-κB pathways. In periodontitis-affected mice, 8AGPB reduces inflammatory infiltrate, osteoclastic activity, and bone resorption.^285^
- Ginsenoside Rb3, tested in LPS-induced periodontitis, significantly downregulates osteoclast markers (NFATc1, MMP9, Cathepsin K, Acp5), suppresses p38 MAPK, ERK, and NF-κB signaling, and decreases p-ERK expression in gingival tissues after Rb3 treatment.^286^
- The JNK inhibitor SP600125 reduces cytokine expression and bone resorption in arthritis models,^287^ and when delivered via carrier systems, regulates both JNK and JAK-STAT pathways, diminishing osteoclastogenesis and protecting joint integrity.^288^
- ERK inhibitors such as PD98059, which block MEK activation, reduce ERK phosphorylation and pro-inflammatory cytokine expression in models of hyperglycemia-associated periodontitis.^289^ However, further clinical validation is required, as off-target effects remain a concern.
- PF-3845, a FAAH inhibitor, suppresses MEK, ERK, and IκBα phosphorylation, reducing NFATc1 expression, osteoclast activity, and bone resorption, in a ligature-induced periodontitis model.^290^ Similarly, chitooligosaccharide (CS) inhibits MAPK activation and osteoclast-related gene expression both in vitro and in vivo, effectively preventing bone loss in ligature-induced periodontitis.^148^

In summary, p38, JNK, and ERK MAPKs are key mediators of inflammation and bone loss in periodontitis. Targeting these pathways with inhibitors or natural compounds offers promising strategies to mitigate disease progression. However, the potential for off-target effects and safety concerns has limited clinical translation.^38,291–293^ Continued investigation is required to develop more selective and effective MAPK-targeted therapies for inflammatory bone diseases.

### Therapeutic strategies targeting the PI3K/Akt signaling pathway

**Fig. 15:**
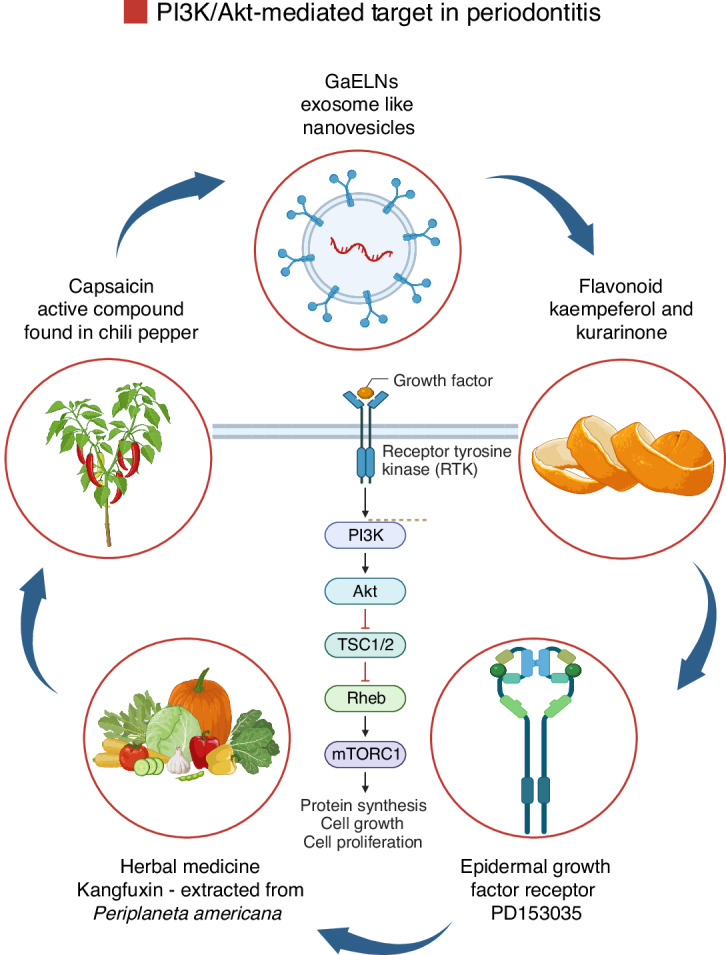
Therapeutic Strategies Targeting the PI3K/Akt Signaling Pathway. Several therapeutic strategies have been explored to modulate bone resorption by targeting the PI3K/Akt signaling pathway and its downstream effects. These approaches aim to attenuate inflammation and prevent excessive bone loss. They include the use of PI3K/Akt pathway inhibitors, natural compounds, growth factors, and exosomes. This figure summarizes and highlights the potential benefits of these therapeutic interventions in the context of periodontitis. Created with BioRender.com (License Number: EQ28PGD0KN)

Considering the pivotal involvement of PI3K/Akt signaling in inflammation and osteoclast differentiation associated with periodontitis, various therapeutic approaches have been investigated (Fig. 15)

- GaELNs, a garlic-derived exosome-like nanovesicle, significantly suppresses pro-inflammatory cytokine and ROS levels production in vitro. In the mouse periodontitis model, local administration of GaELNs significantly reduces gingival inflammation and alveolar bone resorption, and decreases the expression of inflammatory mediators (TNF-α, IL-12, CXCL-12, and CXCL-5) through activation of the PHGDH/PI3K/Akt signaling pathway. Its upregulation enhances PI3K/AKT phosphorylation and downstream activation of mTOR, VEGF, and Nrf2, while suppressing NF-κB activity.^202^ These findings support the potential application of GaELNs as a safe, effective, and plant-based nanotherapy for inflammatory periodontal diseases.
- Kaempferol, a natural flavonol, restores osteogenic capacity in LPS-treated PDLSCs, enhancing ALP activity, calcium deposition, and expression of osteogenesis-related genes and proteins through activation of the PI3K/Akt and P38 MAPK pathways.^294^ In a murine ligature-induced periodontitis model, kaempferol (10 mg/kg) significantly reduces alveolar bone loss (by ~63.8%), improve bone volume/tissue volume ratio and increased expression of OPN, EphrinB2, p-PI3K, p-Akt, and p-P38 in periodontal tissues of kaempferol-treated mice. These molecular and structural effects collectively confirmed that kaempferol promotes periodontal bone regeneration by targeting EphrinB2 and activating the PI3K/Akt and P38 signaling pathways.^294^
- Kurarinone (KR), a flavonoid derived from *Sophora flavescens*, significantly decreases the differentiation and function of osteoclasts derived from BMM, and inhibit the PI3K/AKT/GSK-3β signaling axis.^199^ Using a mouse model of LPS-induced bone resorption, KR treatment significantly reduces bone loss, osteoclast numbers, and bone surface erosion. KR effectively mitigates inflammatory bone loss by inhibiting osteoclast differentiation and function, suppressing oxidative stress, and downregulating inflammatory mediators.^199^ These effects are primarily mediated through the activation of the Nrf2 antioxidant pathway and inhibition of the PI3K/AKT signaling cascade.
- PD153035, an epidermal growth factor receptor (EGFR) inhibitor, was evaluated in a periodontitis mouse model. Fibulin-3 (FBLN3), a secreted extracellular matrix (ECM) glycoprotein, was shown to bind to EGFR, leading to activation of the PI3K/AKT signaling pathway, which is essential for its pro-M1 macrophage effects. Using the PD153035 inhibitor reverses FBLN3-induced activation of PI3K/AKT and mitigate M1 polarization, significantly reduces alveolar bone loss, decreases local expression of inflammatory cytokines (IL-1β and IL-6), and shift macrophage populations toward the anti-inflammatory M2 phenotype.^295^
- Kangfuxin, a traditional Chinese medicine extract derived from *Periplaneta americana*, significantly attenuates key pro-inflammatory cytokines (IL-6, IL-1β, and TNF-α) at both the mRNA and protein levels in a rat periodontitis model. Histological analysis further reveals reduced leukocyte infiltration and better preservation of alveolar bone and cementum structure compared to untreated periodontitis controls. Kangfuxin inhibits phosphorylation of PI3K/Akt and mTOR, indicating suppression of the PI3K/Akt/mTOR signaling pathway. The therapeutic effects of this fraction is abolished when a PI3K activator was co-administered, confirming that modulation of this pathway is central to its activity.^200^
- Capsaicin, an active component found in chili peppers, significantly promotes the proliferation, migration, and osteogenic differentiation of PDLSCs, as evidenced by increased ALP activity, mineralization, and expression of osteogenic markers (Runx2 and ALP) induced by *P. gingivalis*. Capsaicin activated the PI3K/AKT/mTOR signaling pathway, enhancing the phosphorylation of key proteins (PI3K, AKT, and mTOR). This activation was linked to the upregulation of osteogenic differentiation. In a mouse periodontitis model, capsaicin pretreatment significantly reduces alveolar bone loss and increases the expression of osteogenic proteins (ALP and Runx2), demonstrating its protective role against inflammation-induced bone resorption.^201^

## Concluding remarks and future directions

Although periodontitis is characterized by progressive alveolar bone loss, longitudinal clinical and histological studies have consistently demonstrated that this tissue destruction does not occur in a continuous linear fashion. Instead, the disease typically follows an episodic pattern marked by bursts of activity interspersed with periods of quiescence or remission.^296,297^ This fluctuating course poses a critical challenge when translating therapeutic interventions from animal models to human clinical application. In contrast to human disease, experimental animal models of periodontitis, particularly those involving ligature placement, bacterial inoculation or LPS injections, tend to induce acute and synchronized osteoclastic activity over relatively short durations (e.g., 7–21 days), which does not fully recapitulate the chronic, intermittent progression observed in clinical settings.^222,224,225,298^

The timing and delivery of therapeutic agents, especially small molecules and biologics, must therefore be strategically optimized to align with the underlying pathophysiological dynamics of human periodontitis. Current clinical tools do not adequately detect early disease activation or predict exacerbations, nor are there reliable biomarkers to distinguish between active and quiescent phases, which could further limit research models. The identification of some biomarkers, such as elevated levels of active MMP-8 or IL-1β in gingival crevicular fluid or saliva, may enable precision-targeted therapy during periods of heightened osteoclastic activity.^299,300^ This targeted approach could help minimize unnecessary exposure to expensive or potentially immunosuppressive biologics, such as anti-TNF-α or anti-RANKL monoclonal antibodies, outside of periods of active bone resorption. Moreover, site-specific delivery platforms, such as injectable hydrogels, bioresorbable microspheres, or mucoadhesive gels, are emerging as promising strategies to localize drug action while reducing systemic side effects.^249,301–303^

Given the complex etiology and chronicity of periodontitis, future research should focus on integrating molecular diagnostics, real-time disease activity monitoring, and patient stratification to guide personalized therapy. Studies are needed to (1) define molecular signatures predictive of disease activation, (2) develop controlled-release systems tailored for oral environments, and (3) evaluate the long-term safety and cost-effectiveness of these interventions in well-designed clinical trials. Moreover, leveraging multi-omics approaches, including transcriptomics, proteomics, and metabolomics, could aid in identifying patient subtypes that are more likely to benefit from biologic therapies versus adjunctive natural compounds or host-modulating agents.

The use of dietary supplements such as flavonoids, probiotics, or other herbal medicine may offer supportive benefits due to their favorable safety profile. Despite their promising biological activities, the clinical relevance of these natural compounds remains uncertain due to some limitations.^304,305^ Most human studies evaluating their efficacy have been of short duration, involved small sample sizes, and often lacked rigorous design. Moreover, bioavailability is a major concern, as many of these natural compounds have poor systemic absorption, rapid metabolism, and low tissue penetration when administered orally, thereby limiting their therapeutic concentrations at periodontal sites.^306,307^ While they are generally safe and well-tolerated as dietary supplements,^304–307^ their effects are often modest and unlikely to produce clinically meaningful improvements in the absence of conventional periodontal therapy. Therefore, although natural compounds may serve as supportive agents for maintaining periodontal health, particularly in individuals with systemic inflammation or at risk for disease progression, their stand-alone impact on arresting or reversing periodontitis remains questionable without robust, large-scale clinical validation. The translation of findings from acute animal models to the episodic nature of human periodontitis necessitates a precision medicine framework that incorporates temporal disease patterns, biomarker surveillance, and advanced delivery technologies.

In summary, understanding the signaling pathways involved in inflammatory diseases such as periodontitis is critical for developing targeted therapies that mitigate disease progression and tissue destruction. Central to this pathological process is the activation of intracellular signaling pathways triggered by microbial and inflammatory cues. Among these, the NF-κB signaling pathway coordinates immune responses and osteoclast differentiation. Its activation in periodontal tissues in response to microbial stimuli amplifies inflammation and bone resorption. Several therapeutic approaches have been proposed to target NF-κB signaling:

- Small molecule inhibitors offer direct suppression of NF-κB activation, attenuating inflammatory cytokine production and osteoclastogenesis. While promising, their clinical translation requires further validation regarding specificity and safety.
- Natural compounds, such as curcumin, resveratrol, tanshinone, and hesperetin, exhibit anti-inflammatory and anti-resorptive properties through NF-κB inhibition, offering biocompatible alternatives or adjuncts to conventional therapy.
- Biologic agents, including IL-1, IL-6, and TNF-α inhibitors, have demonstrated remarkable efficacy in reducing systemic and local inflammation and preserving alveolar bone. However, their high cost and potential long-term effects on oral immunity warrant cautious use in periodontitis management.
- Targeting the RANK-RANKL-OPG axis, with agents such as denosumab, directly disrupts osteoclastogenesis and bone resorption. Nonetheless, these therapies must be administered with care due to risks such as medication-related osteonecrosis of the jaw.

The JAK/STAT pathway regulates key aspects of immune cell activation, survival, and cytokine signaling. Its persistent activation contributes to chronic inflammation and bone loss in periodontitis and related diseases. JAK inhibitors, STAT3 inhibitors, and cytokine antagonists have shown therapeutic potential, yet further clinical trials are essential to determine their long-term safety and efficacy in oral inflammatory contexts.

Similarly, the MAPK signaling pathway, including p38 MAPK, JNK, and ERK cascades, governs cellular responses to inflammatory stress and microbial stimuli. Its activation promotes cytokine release and osteoclast differentiation, reinforcing periodontal tissue destruction. Therapeutic agents targeting MAPKs have shown efficacy in preclinical models; however, challenges such as off-target effects and systemic toxicity have hindered clinical advancement. Precision-targeted MAPK inhibitors, alongside delivery systems that enhance local bioavailability, may overcome these limitations.

The PI3K)/Akt signaling pathway is a critical intracellular cascade involved in regulating cell survival, proliferation, metabolism, and immune responses. Upon activation by RTKs, cytokine receptors, or G protein-coupled receptors, PI3K catalyzes the conversion of PIP2 to PIP3, leading to the recruitment and activation of Akt (protein kinase B). Activated Akt phosphorylates a wide range of downstream targets to promote cell growth and survival, while inhibiting apoptotic pathways. Dysregulation of PI3K/Akt signaling is implicated in various pathological conditions, including periodontitis and its modulation have shown efficacy in inhibiting alveolar bone loss during periodontitis progression.

In conclusion, signaling pathways such as NF-κB, JAK/STAT, and MAPK represent promising therapeutic targets for controlling inflammation and bone resorption in periodontitis, at least in animal models of induced periodontitis. Advances in small molecule inhibitors, natural compounds, biologics, and pathway-specific strategies continue to expand the therapeutic landscape. Future research and clinical trials should optimize efficacy, minimize adverse effects, and integrate these approaches into personalized treatment strategies for periodontitis and other inflammatory bone disorders.

## Data Availability

No original data were used in this manuscript.

## References

[1] Hienz, S. A., Paliwal, S. & Ivanovski, S. Mechanisms of bone resorption in periodontitis. *J. Immunol. Res.* **2015**, 615486 (2015). 26065002 10.1155/2015/615486 PMC4433701

[2] de Molon, R. S., de Avila, E. D., Cirelli, J. A. & Steffens, J. P. Periodontal research contributions to basic sciences: from cell communication and host-parasite interactions to inflammation and bone biology. *Biocell* **46**, 633–638 (2022).

[3] Caton, J. G. et al. A new classification scheme for periodontal and peri-implant diseases and conditions—introduction and key changes from the 1999 classification. *J. Clin. Periodontol.* **45**, S1–S8 (2018). 29926489 10.1111/jcpe.12935

[4] Hajishengallis, G. Periodontitis: from microbial immune subversion to systemic inflammation. *Nat. Rev. Immunol.* **15**, 30–44 (2015). 25534621 10.1038/nri3785 PMC4276050

[5] Hajishengallis, G. & Chavakis, T. Local and systemic mechanisms linking periodontal disease and inflammatory comorbidities. *Nat. Rev. Immunol.* **21**, 426–440 (2021). 33510490 10.1038/s41577-020-00488-6 PMC7841384

[6] Kinane, D. F., Lappin, D. F. & Culshaw, S. The role of acquired host immunity in periodontal diseases. *Periodontol*10.1111/prd.12562 (2024). 10.1111/prd.12562 38641953

[7] Pan, W., Wang, Q. & Chen, Q. The cytokine network involved in the host immune response to periodontitis. *Int. J. Oral. Sci.* **11**, 30 (2019). 31685798 10.1038/s41368-019-0064-z PMC6828663

[8] Eke, P. I., Thornton-Evans, G., Dye, B. & Genco, R. Advances in surveillance of periodontitis: the centers for disease control and prevention periodontal disease surveillance project. *J. Periodontol.* **83**, 1337–1342 (2012). 22324489 10.1902/jop.2012.110676 PMC6004792

[9] Kassebaum, N. J. et al. Global burden of severe periodontitis in 1990-2010: a systematic review and meta-regression. *J. Dent. Res*. **93**, 1045–1053 (2014). 25261053 10.1177/0022034514552491 PMC4293771

[10] Eke, P. I. et al. Update on prevalence of periodontitis in adults in the United States: NHANES 2009 to 2012. *J. Periodontol.* **86**, 611–622 (2015). 25688694 10.1902/jop.2015.140520 PMC4460825

[11] Balta, M. G., Papathanasiou, E., Blix, I. J. & Van Dyke, T. E. Host modulation and treatment of periodontal disease. *J. Dent. Res.* **100**, 798–809 (2021). 33655803 10.1177/0022034521995157 PMC8261853

[12] Phipps, K. R. & Stevens, V. J. Relative contribution of caries and periodontal disease in adult tooth loss for an HMO dental population. *J. Public Health Dent.* **55**, 250–252 (1995). 8551465 10.1111/j.1752-7325.1995.tb02377.x

[13] Listl, S., Galloway, J., Mossey, P. A. & Marcenes, W. Global economic impact of dental diseases. *J. Dent. Res.* **94**, 1355–1361 (2015). 26318590 10.1177/0022034515602879

[14] de Aquino, S. G. et al. Periodontal pathogens directly promote autoimmune experimental arthritis by inducing a TLR2- and IL-1-driven Th17 response. *J. Immunol.* **192**, 4103–4111 (2014). 24683190 10.4049/jimmunol.1301970

[15] de Molon, R. S., Rossa, C., Thurlings, R. M., Cirelli, J. A. & Koenders, M. I. Linkage of periodontitis and rheumatoid arthritis: current evidence and potential biological interactions. *Int. J. Mol. Sci* **20**, 4541 (2019). 31540277 10.3390/ijms20184541 PMC6769683

[16] de Aquino, S. G. et al. The aggravation of arthritis by periodontitis is dependent of IL-17 receptor A activation. *J. Clin. Periodontol.* **44**, 881–891 (2017). 28498497 10.1111/jcpe.12743

[17] Gonzalez-Febles, J. & Sanz, M. Periodontitis and rheumatoid arthritis: what have we learned about their connection and their treatment? *Periodontol 2000* **87**, 181–203 (2021). 34463976 10.1111/prd.12385

[18] Shahbaz, M. et al. Connecting the dots: NETosis and the periodontitis-rheumatoid arthritis nexus. *Int. J. Rheum. Dis.* **27**, e15415 (2024). 39526323 10.1111/1756-185X.15415

[19] Belizario, L. C. G. et al. The impact of type 2 diabetes mellitus on non-surgical periodontal treatment: a non-randomized clinical trial. *J. Clin. Med.* **13**, 5978 (2024). 10.3390/jcm13195978 PMC11477662 39408037

[20] de Molon, R. S. et al. The efficacy of topical or systemic antibiotics as adjuvants to non-surgical periodontal treatment in diabetic patients: a systematic review and meta-analysis of randomized clinical trials. *J. Clin. Med.* **13**, 4763 (2024). 10.3390/jcm13164763 PMC11355856 39200907

[21] Lalla, E. & Papapanou, P. N. Diabetes mellitus and periodontitis: a tale of two common interrelated diseases. *Nat. Rev. Endocrinol.* **7**, 738–748 (2011). 21709707 10.1038/nrendo.2011.106

[22] Wu, C. Z. et al. Epidemiologic relationship between periodontitis and type 2 diabetes mellitus. *BMC Oral. Health* **20**, 204 (2020). 32652980 10.1186/s12903-020-01180-w PMC7353775

[23] Rodrigues, J. V. S. et al. The effect of non-surgical periodontal treatment on patients with combined refractory arterial hypertension and stage III, Grade B periodontitis: a preliminary prospective clinical study. *J. Clin. Med.* **12**, 4277 (2023). 10.3390/jcm12134277 PMC10342463 37445313

[24] Rosa, R. A. C. et al. The relationship between hypertension and periodontitis: a cross-sectional study. *J. Clin. Med.* **12**, 5140 (2023). 10.3390/jcm12155140 PMC10419474 37568542

[25] Khumaedi, A. I., Purnamasari, D., Wijaya, I. P. & Soeroso, Y. The relationship of diabetes, periodontitis and cardiovascular disease. *Diab. Metab. Syndr.* **13**, 1675–1678 (2019). 10.1016/j.dsx.2019.03.023 31336540

[26] Liccardo, D. et al. Periodontal disease: a risk factor for diabetes and cardiovascular disease. *Int. J. Mol. Sci*. **20**, 1414 (2019). 10.3390/ijms20061414 PMC6470716 30897827

[27] Sanz, M. et al. Periodontitis and cardiovascular diseases: consensus report. *J. Clin. Periodontol.* **47**, 268–288 (2020). 32011025 10.1111/jcpe.13189 PMC7027895

[28] Chapple, I. L. C., Hirschfeld, J., Cockwell, P., Dietrich, T. & Sharma, P. Interplay between periodontitis and chronic kidney disease. *Nat. Rev. Nephrol.* **21**, 226–240 (2025). 39658571 10.1038/s41581-024-00910-5

[29] Kuraji, R., Sekino, S., Kapila, Y. & Numabe, Y. Periodontal disease-related nonalcoholic fatty liver disease and nonalcoholic steatohepatitis: an emerging concept of oral-liver axis. *Periodontol 2000* **87**, 204–240 (2021). 34463983 10.1111/prd.12387 PMC8456799

[30] Baima, G. et al. Periodontitis and risk of cancer: mechanistic evidence. *Periodontol 2000* **96**, 83–94 (2024). 38102837 10.1111/prd.12540 PMC11579815

[31] Zhou, Y., Meyle, J. & Groeger, S. Periodontal pathogens and cancer development. *Periodontol 2000* **96**, 112–149 (2024). 38965193 10.1111/prd.12590 PMC11579836

[32] Beck, J. D., Papapanou, P. N., Philips, K. H. & Offenbacher, S. Periodontal medicine: 100 years of progress. *J. Dent. Res.* **98**, 1053–1062 (2019). 31429666 10.1177/0022034519846113

[33] Theodoro, L. H. et al. Role of junctional epithelium in maintaining dento-gingival adhesion and periodontal health. *Front. Dent. Med.* **4**, 1144537 (2023). 39916903 10.3389/fdmed.2023.1144537 PMC11797802

[34] Gomes, N. A. et al*.* Expression and localization of amelotin, laminin, and protein secreted by follicular dendritic cells after ligature-induced experimental periodontitis in rats. *Br. Dent. J.*10.1038/s41415-025-8508-7. (2025). 10.1038/s41415-025-8508-7 40691709

[35] Hajishengallis, G., Chavakis, T. & Lambris, J. D. Current understanding of periodontal disease pathogenesis and targets for host-modulation therapy. *Periodontol 2000* **84**, 14–34 (2020). 32844416 10.1111/prd.12331 PMC7457922

[36] Abdulkareem, A. A. et al. Current concepts in the pathogenesis of periodontitis: from symbiosis to dysbiosis. *J. Oral. Microbiol.* **15**, 2197779 (2023). 37025387 10.1080/20002297.2023.2197779 PMC10071981

[37] Kirkwood, K. L., Cirelli, J. A., Rogers, J. E. & Giannobile, W. V. Novel host response therapeutic approaches to treat periodontal diseases. *Periodontol 2000* **43**, 294–315 (2007). 17214846 10.1111/j.1600-0757.2006.00166.x PMC2570321

[38] Kirkwood, K. L. & Rossa, C. Jr The potential of p38 MAPK inhibitors to modulate periodontal infections. *Curr. Drug Metab.* **10**, 55–67 (2009). 19149513 10.2174/138920009787048347 PMC2810486

[39] Souza, J. A., Rossa, C. Jr, Garlet, G. P., Nogueira, A. V. & Cirelli, J. A. Modulation of host cell signaling pathways as a therapeutic approach in periodontal disease. *J. Appl. Oral. Sci.* **20**, 128–138 (2012). 22666826 10.1590/S1678-77572012000200002 PMC3894752

[40] Boyce, B. F. & Xing, L. Functions of RANKL/RANK/OPG in bone modeling and remodeling. *Arch. Biochem. Biophys.* **473**, 139–146 (2008). 18395508 10.1016/j.abb.2008.03.018 PMC2413418

[41] De Leon-Oliva, D. et al. The RANK-RANKL-OPG system: a multifaceted regulator of homeostasis, immunity, and cancer. *Medicina* **59**, 1752 (2023). 37893470 10.3390/medicina59101752 PMC10608105

[42] Di Cicco, G. et al. The pathogenetic role of RANK/RANKL/OPG signaling in osteoarthritis and related targeted therapies. *Biomedicines* **12**, 2292 (2024). 39457605 10.3390/biomedicines12102292 PMC11505501

[43] Silva, N. et al. Host response mechanisms in periodontal diseases. *J. Appl. Oral. Sci.* **23**, 329–355 (2015). 26221929 10.1590/1678-775720140259 PMC4510669

[44] Hajishengallis, G. Immunomicrobial pathogenesis of periodontitis: keystones, pathobionts, and host response. *Trends Immunol.* **35**, 3–11 (2014). 24269668 10.1016/j.it.2013.09.001 PMC3947349

[45] Jakubovics, N. S., Goodman, S. D., Mashburn-Warren, L., Stafford, G. P. & Cieplik, F. The dental plaque biofilm matrix. *Periodontol 2000* **86**, 32–56 (2021). 33690911 10.1111/prd.12361 PMC9413593

[46] Graves, D. T., Li, J. & Cochran, D. L. Inflammation and uncoupling as mechanisms of periodontal bone loss. *J. Dent. Res.* **90**, 143–153 (2011). 21135192 10.1177/0022034510385236 PMC3144100

[47] Isola, G. et al. Periodontal health and disease in the context of systemic diseases. *Mediators Inflamm.* **2023**, 9720947 (2023). 37214190 10.1155/2023/9720947 PMC10199803

[48] Sirisereephap, K. et al. Osteoimmunology in periodontitis: local proteins and compounds to alleviate periodontitis. *Int. J. Mol. Sci.* **23**, 5540 (2022). 10.3390/ijms23105540 PMC9146968 35628348

[49] Valverde, A., George, A., Nares, S. & Naqvi, A. R. Emerging therapeutic strategies targeting bone signaling pathways in periodontitis. *J. Periodontal Res.* **60**, 101–120 (2025). 39044454 10.1111/jre.13326 PMC11873684

[50] Usui, M. et al. Mechanism of alveolar bone destruction in periodontitis—periodontal bacteria and inflammation. *Jpn Dent. Sci. Rev.* **57**, 201–208 (2021). 34703508 10.1016/j.jdsr.2021.09.005 PMC8524191

[51] Hajishengallis, G., Darveau, R. P. & Curtis, M. A. The keystone-pathogen hypothesis. *Nat. Rev. Microbiol.* **10**, 717–725 (2012). 22941505 10.1038/nrmicro2873 PMC3498498

[52] Kinane, D. F. & Lappin, D. F. Immune processes in periodontal disease: a review. *Ann. Periodontol.* **7**, 62–71 (2002). 16013218 10.1902/annals.2002.7.1.62

[53] He, H., Hao, Y., Fan, Y., Li, B. & Cheng, L. The interaction between innate immunity and oral microbiota in oral diseases. *Expert Rev. Clin. Immunol.* **19**, 405–415 (2023). 36803467 10.1080/1744666X.2023.2182291

[54] Hajishengallis, G. & Diaz, P. I. Porphyromonas gingivalis: immune subversion activities and role in periodontal dysbiosis. *Curr. Oral. Health Rep.* **7**, 12–21 (2020). 33344104 10.1007/s40496-020-00249-3 PMC7747940

[55] Cekici, A., Kantarci, A., Hasturk, H. & Van Dyke, T. E. Inflammatory and immune pathways in the pathogenesis of periodontal disease. *Periodontol 2000* **64**, 57–80 (2014). 24320956 10.1111/prd.12002 PMC4500791

[56] Gu, Y. & Han, X. Toll-like receptor signaling and immune regulatory lymphocytes in periodontal disease. *Int. J. Mol. Sci.* **21**, 3329 (2020). 10.3390/ijms21093329 PMC7247565 32397173

[57] Papayannopoulos, V. Neutrophil extracellular traps in immunity and disease. *Nat. Rev. Immunol.* **18**, 134–147 (2018). 28990587 10.1038/nri.2017.105

[58] Castanheira, F. V. S. & Kubes, P. Neutrophils and NETs in modulating acute and chronic inflammation. *Blood* **133**, 2178–2185 (2019). 30898862 10.1182/blood-2018-11-844530

[59] Mousa, A. O., Al Hussaini, A. H. A. & Hussein, H. M. The potential role of reactive oxygen species produced by low-density neutrophils in periodontitis. *Eur. J. Dent.* **18**, 1142–1148 (2024). 38744332 10.1055/s-0044-1782211 PMC11479733

[60] Gysemans, C., Beya, M., Pedace, E. & Mathieu, C. Exploring neutrophil heterogeneity and plasticity in health and disease. *Biomedicines* **13**, 597 (2025). 10.3390/biomedicines13030597 PMC11940349 40149573

[61] Moutsopoulos, N. M. & Konkel, J. E. Tissue-specific immunity at the oral mucosal barrier. *Trends Immunol.* **39**, 276–287 (2018). 28923364 10.1016/j.it.2017.08.005 PMC5843496

[62] Kantarci, A., Oyaizu, K. & Van Dyke, T. E. Neutrophil-mediated tissue injury in periodontal disease pathogenesis: findings from localized aggressive periodontitis. *J. Periodontol.* **74**, 66–75 (2003). 12593599 10.1902/jop.2003.74.1.66

[63] Dutzan, N. et al. A dysbiotic microbiome triggers T(H)17 cells to mediate oral mucosal immunopathology in mice and humans. *Sci. Transl. Med.* **10**, eaat0797 (2018). 10.1126/scitranslmed.aat0797 PMC6330016 30333238

[64] Hajishengallis, G. & Lamont, R. J. Breaking bad: manipulation of the host response by Porphyromonas gingivalis. *Eur. J. Immunol.* **44**, 328–338 (2014). 24338806 10.1002/eji.201344202 PMC3925422

[65] Chapple, I. L. C., Hirschfeld, J., Kantarci, A., Wilensky, A. & Shapira, L. The role of the host-Neutrophil biology. *Periodontol**2000*. 10.1111/prd.12490 (2023). 10.1111/prd.12490 37199393

[66] Konig, M. F. & Andrade, F. A critical reappraisal of neutrophil extracellular traps and NETosis mimics based on differential requirements for protein citrullination. *Front Immunol.* **7**, 461 (2016). 27867381 10.3389/fimmu.2016.00461 PMC5095114

[67] White, P. C., Chicca, I. J., Cooper, P. R., Milward, M. R. & Chapple, I. L. Neutrophil extracellular traps in periodontitis: a web of intrigue. *J. Dent. Res.* **95**, 26–34, (2016). 26442948 10.1177/0022034515609097

[68] Divangahi, M. et al. Trained immunity, tolerance, priming and differentiation: distinct immunological processes. *Nat. Immunol.* **22**, 2–6 (2021). 33293712 10.1038/s41590-020-00845-6 PMC8020292

[69] Li, X. et al. Maladaptive innate immune training of myelopoiesis links inflammatory comorbidities. *Cell* **185**, 1709–1727.e1718 (2022). 35483374 10.1016/j.cell.2022.03.043 PMC9106933

[70] Haacke, N. et al. Innate immune training of osteoclastogenesis promotes inflammatory bone loss in mice. *Dev. Cell* **60**, 1854–1870 (2025). 40020679 10.1016/j.devcel.2025.02.001 PMC7617534

[71] Mo, K., Wang, Y., Lu, C. & Li, Z. Insight into the role of macrophages in periodontitis restoration and development. *Virulence* **15**, 2427234 (2024). 39535076 10.1080/21505594.2024.2427234 PMC11572313

[72] Sun, X. et al. Polarized macrophages in periodontitis: characteristics, function, and molecular signaling. *Front. Immunol.* **12**, 763334 (2021). 34950140 10.3389/fimmu.2021.763334 PMC8688840

[73] Kini, V., Mohanty, I., Telang, G. & Vyas, N. Immunopathogenesis and distinct role of Th17 in periodontitis: a review. *J. Oral. Biosci.* **64**, 193–201 (2022). 35489583 10.1016/j.job.2022.04.005

[74] Bunte, K. & Beikler, T. Th17 Cells and the IL-23/IL-17 axis in the pathogenesis of periodontitis and immune-mediated inflammatory diseases. *Int. J. Mol. Sci.* **20**, 3394 (2019). 31295952 10.3390/ijms20143394 PMC6679067

[75] Gaffen, S. L. & Hajishengallis, G. A new inflammatory cytokine on the block: re-thinking periodontal disease and the Th1/Th2 paradigm in the context of Th17 cells and IL-17. *J. Dent. Res.* **87**, 817–828 (2008). 18719207 10.1177/154405910808700908 PMC2692983

[76] Rastogi, I. et al. Role of B cells as antigen presenting cells. *Front. Immunol.* **13**, 954936 (2022). 36159874 10.3389/fimmu.2022.954936 PMC9493130

[77] de Gruijter, N. M., Jebson, B. & Rosser, E. C. Cytokine production by human B cells: role in health and autoimmune disease. *Clin. Exp. Immunol.* **210**, 253–262 (2022). 36179248 10.1093/cei/uxac090 PMC9985175

[78] Huang, J., Cai, X., Ou, Y., Zhou, Y. & Wang, Y. Resolution of inflammation in periodontitis: a review. *Int. J. Clin. Exp. Pathol.* **11**, 4283–4295 (2018). 31949825 PMC6962983

[79] Van Dyke, T. E. & Sima, C. Understanding resolution of inflammation in periodontal diseases: Is chronic inflammatory periodontitis a failure to resolve?. *Periodontol 2000* **82**, 205–213 (2020). 31850636 10.1111/prd.12317

[80] Eltay, E. G. & Van Dyke, T. Resolution of inflammation in oral diseases. *Pharm. Ther.* **247**, 108453 (2023). 10.1016/j.pharmthera.2023.108453 37244405

[81] Mizraji, G., Heyman, O., Van Dyke, T. E. & Wilensky, A. Resolvin D2 restrains Th1 immunity and prevents alveolar bone loss in murine periodontitis. *Front. Immunol.* **9**, 785 (2018). 29922275 10.3389/fimmu.2018.00785 PMC5996935

[82] Sahni, V. & Van Dyke, T. E. Immunomodulation of periodontitis with SPMs. *Front. Oral. Health* **4**, 1288722 (2023). 37927821 10.3389/froh.2023.1288722 PMC10623003

[83] Serhan, C. N. Pro-resolving lipid mediators are leads for resolution physiology. *Nature* **510**, 92–101 (2014). 24899309 10.1038/nature13479 PMC4263681

[84] Van Dyke, T. E. Pro-resolving mediators in the regulation of periodontal disease. *Mol. Asp. Med.* **58**, 21–36 (2017). 10.1016/j.mam.2017.04.006 PMC5660638 28483532

[85] Freire, M. O. & Van Dyke, T. E. Natural resolution of inflammation. *Periodontol 2000* **63**, 149–164 (2013). 23931059 10.1111/prd.12034 PMC4022040

[86] Huang, N. & Gibson, F. C. 3rd. Immuno-pathogenesis of periodontal disease: current and emerging paradigms. *Curr. Oral. Health Rep.* **1**, 124–132 (2014). 24839590 10.1007/s40496-014-0017-8 PMC4020182

[87] Sima, C. & Glogauer, M. Macrophage subsets and osteoimmunology: tuning of the immunological recognition and effector systems that maintain alveolar bone. *Periodontol 2000* **63**, 80–101 (2013). 23931056 10.1111/prd.12032

[88] Hathaway-Schrader, J. D. & Novince, C. M. Maintaining homeostatic control of periodontal bone tissue. *Periodontol 2000* **86**, 157–187 (2021). 33690918 10.1111/prd.12368 PMC8294452

[89] Zhang, M., Liu, Y., Afzali, H. & Graves, D. T. An update on periodontal inflammation and bone loss. *Front. Immunol.* **15**, 1385436 (2024). 38919613 10.3389/fimmu.2024.1385436 PMC11196616

[90] Ferrara, E. & Mastrocola, F. Pattern recognition receptors in periodontal disease: molecular mechanisms, signaling pathways, and therapeutic implications. *J. Mol. Pathol.* **5**, 497–511 (2024).

[91] Seeman, E. Bone modeling and remodeling. *Crit. Rev. Eukaryot. Gene Expr.* **19**, 219–233 (2009). 19883366 10.1615/critreveukargeneexpr.v19.i3.40

[92] Iniguez-Ariza, N. M. & Clarke, B. L. Bone biology, signaling pathways, and therapeutic targets for osteoporosis. *Maturitas* **82**, 245–255 (2015). 26255682 10.1016/j.maturitas.2015.07.003

[93] Bolamperti, S., Villa, I. & Rubinacci, A. Bone remodeling: an operational process ensuring survival and bone mechanical competence. *Bone Res.* **10**, 48 (2022). 35851054 10.1038/s41413-022-00219-8 PMC9293977

[94] Sabokbar, A., Mahoney, D. J., Hemingway, F. & Athanasou, N. A. Non-canonical (RANKL-independent) pathways of osteoclast differentiation and their role in musculoskeletal diseases. *Clin. Rev. Allergy Immunol.* **51**, 16–26 (2016). 26578261 10.1007/s12016-015-8523-6

[95] Sromova, V., Sobola, D. & Kaspar, P. A brief review of bone cell function and importance. *Cells* **12**, 2576 (2023). 37947654 10.3390/cells12212576 PMC10648520

[96] Daponte, V., Henke, K. & Drissi, H. Current perspectives on the multiple roles of osteoclasts: mechanisms of osteoclast-osteoblast communication and potential clinical implications. *Elife* **13**, e95083 (2024). 38591777 10.7554/eLife.95083 PMC11003748

[97] Kim, J. M., Lin, C., Stavre, Z., Greenblatt, M. B. & Shim, J. H. Osteoblast-osteoclast communication and bone homeostasis. *Cells* **9**, 2073 (2020). 32927921 10.3390/cells9092073 PMC7564526

[98] Hascoet, E. et al. New insights into inflammatory osteoclast precursors as therapeutic targets for rheumatoid arthritis and periodontitis. *Bone Res.* **11**, 26 (2023). 37217496 10.1038/s41413-023-00257-w PMC10203317

[99] Xu, H. et al. Targeting strategies for bone diseases: signaling pathways and clinical studies. *Signal Transduct. Target Ther.* **8**, 202 (2023). 37198232 10.1038/s41392-023-01467-8 PMC10192458

[100] Feng, X. & Teitelbaum, S. L. Osteoclasts: new insights. *Bone Res.* **1**, 11–26 (2013). 26273491 10.4248/BR201301003 PMC4472093

[101] Veis, D. J. & O’Brien, C. A. Osteoclasts, master sculptors of bone. *Annu. Rev. Pathol.* **18**, 257–281 (2023). 36207010 10.1146/annurev-pathmechdis-031521-040919

[102] Grewe, J. M. et al. The role of sphingosine-1-phosphate in bone remodeling and osteoporosis. *Bone Res.* **10**, 34 (2022). 35396384 10.1038/s41413-022-00205-0 PMC8993882

[103] Soe, K., Delaisse, J. M. & Borggaard, X. G. Osteoclast formation at the bone marrow/bone surface interface: importance of structural elements, matrix, and intercellular communication. *Semin. Cell Dev. Biol.* **112**, 8–15 (2021). 32563679 10.1016/j.semcdb.2020.05.016

[104] Ishii, M. et al. Sphingosine-1-phosphate mobilizes osteoclast precursors and regulates bone homeostasis. *Nature* **458**, 524–528 (2009). 19204730 10.1038/nature07713 PMC2785034

[105] Theill, L. E., Boyle, W. J. & Penninger, J. M. RANK-L and RANK: T cells, bone loss, and mammalian evolution. *Annu. Rev. Immunol.* **20**, 795–823 (2002). 11861618 10.1146/annurev.immunol.20.100301.064753

[106] Xiong, J. et al. Matrix-embedded cells control osteoclast formation. *Nat. Med.* **17**, 1235–1241 (2011). 21909103 10.1038/nm.2448 PMC3192296

[107] Asagiri, M. et al. Autoamplification of NFATc1 expression determines its essential role in bone homeostasis. *J. Exp. Med.* **202**, 1261–1269 (2005). 16275763 10.1084/jem.20051150 PMC2213228

[108] Boyle, W. J., Simonet, W. S. & Lacey, D. L. Osteoclast differentiation and activation. *Nature* **423**, 337–342 (2003). 12748652 10.1038/nature01658

[109] Takayanagi, H. et al. Induction and activation of the transcription factor NFATc1 (NFAT2) integrate RANKL signaling in terminal differentiation of osteoclasts. *Dev. Cell* **3**, 889–901 (2002). 12479813 10.1016/s1534-5807(02)00369-6

[110] Vaananen, H. K. & Horton, M. The osteoclast clear zone is a specialized cell-extracellular matrix adhesion structure. *J. Cell Sci.* **108**, 2729–2732 (1995). 7593313 10.1242/jcs.108.8.2729

[111] Nesbitt, S. A. & Horton, M. A. Trafficking of matrix collagens through bone-resorbing osteoclasts. *Science* **276**, 266–269 (1997). 9092478 10.1126/science.276.5310.266

[112] Takegahara, N., Kim, H. & Choi, Y. Unraveling the intricacies of osteoclast differentiation and maturation: insight into novel therapeutic strategies for bone-destructive diseases. *Exp. Mol. Med.* **56**, 264–272 (2024). 38297158 10.1038/s12276-024-01157-7 PMC10907717

[113] Vaananen, H. K., Zhao, H., Mulari, M. & Halleen, J. M. The cell biology of osteoclast function. *J. Cell Sci.* **113**, 377–381 (2000). 10639325 10.1242/jcs.113.3.377

[114] Ajani, J. A., Levin, B. & Wallace, S. Systemic and regional therapy of advanced islet cell tumors. *Gastroenterol. Clin. North Am.* **18**, 923–930 (1989). 2559037

[115] Hartman, G. D. & Duggan, M. E. alpha(v)beta(3) Integrin antagonists as inhibitors of bone resorption. *Expert Opin. Investig. Drugs* **9**, 1281–1291 (2000). 11060743 10.1517/13543784.9.6.1281

[116] Guasto, A. & Cormier-Daire, V. Signaling pathways in bone development and their related skeletal dysplasia. *Int. J. Mol. Sci* **22**, 4321 (2021). 33919228 10.3390/ijms22094321 PMC8122623

[117] Capulli, M., Paone, R. & Rucci, N. Osteoblast and osteocyte: games without frontiers. *Arch. Biochem. Biophys.* **561**, 3–12 (2014). 24832390 10.1016/j.abb.2014.05.003

[118] Matsuo, K. & Irie, N. Osteoclast-osteoblast communication. *Arch. Biochem. Biophys.* **473**, 201–209 (2008). 18406338 10.1016/j.abb.2008.03.027

[119] Knothe Tate, M. L., Adamson, J. R., Tami, A. E. & Bauer, T. W. The osteocyte. *Int. J. Biochem. Cell Biol.* **36**, 1–8 (2004). 14592527 10.1016/s1357-2725(03)00241-3

[120] Bonewald, L. F. The amazing osteocyte. *J. Bone Miner. Res.* **26**, 229–238 (2011). 21254230 10.1002/jbmr.320 PMC3179345

[121] McDonald, M. M. et al. Osteoclasts recycle via osteomorphs during RANKL-stimulated bone resorption. *Cell* **184**, 1330–1347.e1313 (2021). 33636130 10.1016/j.cell.2021.02.002 PMC7938889

[122] Lassen, N. E. et al. Coupling of bone resorption and formation in real time: new knowledge gained from human Haversian BMUs. *J. Bone Miner. Res.* **32**, 1395–1405 (2017). 28177141 10.1002/jbmr.3091

[123] Kohli, S. S. & Kohli, V. S. Role of RANKL-RANK/osteoprotegerin molecular complex in bone remodeling and its immunopathologic implications. *Indian J. Endocrinol. Metab.* **15**, 175–181 (2011). 21897893 10.4103/2230-8210.83401 PMC3156536

[124] Walsh, M. C. & Choi, Y. Biology of the RANKL-RANK-OPG system in immunity, bone, and beyond. *Front. Immunol.* **5**, 511 (2014). 25368616 10.3389/fimmu.2014.00511 PMC4202272

[125] Ono, T., Hayashi, M., Sasaki, F. & Nakashima, T. RANKL biology: bone metabolism, the immune system, and beyond. *Inflamm. Regen.* **40**, 2 (2020). 32047573 10.1186/s41232-019-0111-3 PMC7006158

[126] Takegahara, N., Kim, H. & Choi, Y. RANKL biology. *Bone* **159**, 116353 (2022). 35181574 10.1016/j.bone.2022.116353 PMC9035122

[127] An, J. et al. Natural products for treatment of bone erosive diseases: the effects and mechanisms on inhibiting osteoclastogenesis and bone resorption. *Int. Immunopharmacol.* **36**, 118–131 (2016). 27131574 10.1016/j.intimp.2016.04.024

[128] AlQranei, M. S. & Chellaiah, M. A. Osteoclastogenesis in periodontal diseases: Possible mediators and mechanisms. *J. Oral. Biosci.* **62**, 123–130 (2020). 32081710 10.1016/j.job.2020.02.002 PMC7329621

[129] Kong, Y. Y. et al. OPGL is a key regulator of osteoclastogenesis, lymphocyte development and lymph-node organogenesis. *Nature* **397**, 315–323 (1999). 9950424 10.1038/16852

[130] Dougall, W. C. et al. RANK is essential for osteoclast and lymph node development. *Genes Dev.* **13**, 2412–2424 (1999). 10500098 10.1101/gad.13.18.2412 PMC317030

[131] Mizuno, A. et al. Severe osteoporosis in mice lacking osteoclastogenesis inhibitory factor/osteoprotegerin. *Biochem. Biophys. Res. Commun.* **247**, 610–615 (1998). 9647741 10.1006/bbrc.1998.8697

[132] Simonet, W. S. et al. Osteoprotegerin: a novel secreted protein involved in the regulation of bone density. *Cell* **89**, 309–319 (1997). 9108485 10.1016/s0092-8674(00)80209-3

[133] Ni, S., Shan, F. & Geng, J. Interleukin-10 family members: Biology and role in the bone and joint diseases. *Int. Immunopharmacol.* **108**, 108881 (2022). 35623292 10.1016/j.intimp.2022.108881

[134] Zhao, B. & Ivashkiv, L. B. Negative regulation of osteoclastogenesis and bone resorption by cytokines and transcriptional repressors. *Arthritis Res. Ther.* **13**, 234 (2011). 21861861 10.1186/ar3379 PMC3239342

[135] Yao, Z., Getting, S. J. & Locke, I. C. Regulation of TNF-induced osteoclast differentiation. *Cells* **11**, 132 (2021). 35011694 10.3390/cells11010132 PMC8750957

[136] Zhou, P., Zheng, T. & Zhao, B. Cytokine-mediated immunomodulation of osteoclastogenesis. *Bone* **164**, 116540 (2022). 36031187 10.1016/j.bone.2022.116540 PMC10657632

[137] Chen, S. et al. Macrophages in immunoregulation and therapeutics. *Signal Transduct. Target Ther.* **8**, 207 (2023). 37211559 10.1038/s41392-023-01452-1 PMC10200802

[138] Viniegra, A. et al. Resolving macrophages counter osteolysis by anabolic actions on bone cells. *J. Dent. Res.* **97**, 1160–1169 (2018). 29993312 10.1177/0022034518777973 PMC6169030

[139] Lee, Y. N. et al. c-Fos as a regulator of degranulation and cytokine production in FcepsilonRI-activated mast cells. *J. Immunol.* **173**, 2571–2577 (2004). 15294973 10.4049/jimmunol.173.4.2571

[140] Bozec, A. et al. Fra-2/AP-1 controls bone formation by regulating osteoblast differentiation and collagen production. *J. Cell Biol.* **190**, 1093–1106 (2010). 20837772 10.1083/jcb.201002111 PMC3101588

[141] Malnou, C. E. et al. Heterodimerization with Jun family members regulates c-Fos nucleocytoplasmic traffic. *J. Biol. Chem.* **282**, 31046–31059 (2007). 17681951 10.1074/jbc.M702833200

[142] Matsuo, K. et al. Fosl1 is a transcriptional target of c-Fos during osteoclast differentiation. *Nat. Genet.* **24**, 184–187 (2000). 10655067 10.1038/72855

[143] Wang, Z. Q. et al. Bone and haematopoietic defects in mice lacking c-fos. *Nature* **360**, 741–745 (1992). 1465144 10.1038/360741a0

[144] Grigoriadis, A. E. et al. c-Fos: a key regulator of osteoclast-macrophage lineage determination and bone remodeling. *Science* **266**, 443–448 (1994). 7939685 10.1126/science.7939685

[145] Al Mamun, M. A., Asim, M. M. H., Sahin, M. A. Z. & Al-Bari, M. A. A. Flavonoids compounds from Tridax procumbens inhibit osteoclast differentiation by down-regulating c-Fos activation. *J. Cell Mol. Med.* **24**, 2542–2551, (2020). 31919976 10.1111/jcmm.14948 PMC7028861

[146] Wu, X. et al. Caffeic acid 3,4-dihydroxy-phenethyl ester suppresses receptor activator of NF-kappaB ligand-induced osteoclastogenesis and prevents ovariectomy-induced bone loss through inhibition of mitogen-activated protein kinase/activator protein 1 and Ca^2+^-nuclear factor of activated T-cells cytoplasmic 1 signaling pathways. *J. Bone Miner. Res.* **27**, 1298–1308 (2012). 22337253 10.1002/jbmr.1576

[147] Yang, J. & Peng, B. Correlation between the expression of c-Fos and osteoclasts in induced periapical lesions in rats. *J. Endod.* **34**, 22–25 (2008). 18155486 10.1016/j.joen.2007.07.034

[148] Xu, W. et al. Chitooligosaccharide inhibits RANKL-induced osteoclastogenesis and ligation-induced periodontitis by suppressing MAPK/ c-fos/NFATC1 signaling. *J. Cell Physiol.* **235**, 3022–3032 (2020). 31541460 10.1002/jcp.29207

[149] Ray, N. et al. c-Fos suppresses systemic inflammatory response to endotoxin. *Int. Immunol.* **18**, 671–677 (2006). 16569682 10.1093/intimm/dxl004

[150] Zhang, Z. et al. Pirfenidone inhibits alveolar bone loss in ligature-induced periodontitis by suppressing the NF-kappaB signaling pathway in mice. *Int. J. Mol. Sci.* **24**, 8682 (2023). 37240020 10.3390/ijms24108682 PMC10218211

[151] Jiang, T. et al. Role and regulation of transcription factors in osteoclastogenesis. *Int. J. Mol. Sci.* **24**, 16175 (2023). 38003376 10.3390/ijms242216175 PMC10671247

[152] Kim, J. H. & Kim, N. Regulation of NFATc1 in osteoclast differentiation. *J. Bone Metab.* **21**, 233–241 (2014). 25489571 10.11005/jbm.2014.21.4.233 PMC4255043

[153] Zhao, Q., Shao, J., Chen, W. & Li, Y. P. Osteoclast differentiation and gene regulation. *Front. Biosci.* **12**, 2519–2529 (2007). 17127260 10.2741/2252

[154] Aliprantis, A. O. & Glimcher, L. H. NFATc1 in inflammatory and musculoskeletal conditions. *Adv. Exp. Med. Biol.* **658**, 69–75 (2010). 19950017 10.1007/978-1-4419-1050-9_8

[155] Zhao, Q., Wang, X., Liu, Y., He, A. & Jia, R. NFATc1: functions in osteoclasts. *Int. J. Biochem. Cell Biol.* **42**, 576–579 (2010). 20035895 10.1016/j.biocel.2009.12.018

[156] Greenblatt, M. B. et al. NFATc1 and NFATc2 repress spontaneous osteoarthritis. *Proc. Natl. Acad. Sci. USA* **110**, 19914–19919 (2013). 24248346 10.1073/pnas.1320036110 PMC3856808

[157] Zhong, H., SuYang, H., Erdjument-Bromage, H., Tempst, P. & Ghosh, S. The transcriptional activity of NF-kappaB is regulated by the IkappaB-associated PKAc subunit through a cyclic AMP-independent mechanism. *Cell* **89**, 413–424 (1997). 9150141 10.1016/s0092-8674(00)80222-6

[158] Yamashita, T. et al. NF-kappaB p50 and p52 regulate receptor activator of NF-kappaB ligand (RANKL) and tumor necrosis factor-induced osteoclast precursor differentiation by activating c-Fos and NFATc1. *J. Biol. Chem.* **282**, 18245–18253 (2007). 17485464 10.1074/jbc.M610701200

[159] Souza, P. P. & Lerner, U. H. The role of cytokines in inflammatory bone loss. *Immunol. Investig.* **42**, 555–622 (2013). 24004059 10.3109/08820139.2013.822766

[160] Zhang, C., Yang, L. & Peng, B. Critical role of NFATc1 in periapical lesions. *Int. Endod. J.* **43**, 109–114 (2010). 20078699 10.1111/j.1365-2591.2009.01649.x

[161] Ihn, H. J. et al. A novel benzamide derivative protects ligature-induced alveolar bone erosion by inhibiting NFATc1-mediated osteoclastogenesis. *Toxicol. Appl. Pharm.* **355**, 9–17 (2018). 10.1016/j.taap.2018.06.017 29935282

[162] Kim, H. J. et al. Myeloid-specific PTP1B deficiency attenuates inflammation-induced and ovariectomy-induced bone loss in mice by inhibiting osteoclastogenesis. *J. Bone Miner. Res.* **37**, 505–514 (2022). 34812548 10.1002/jbmr.4478

[163] Da Ponte Leguizamon, N. et al. Phytocystatin CsinCPI-2 reduces osteoclastogenesis and alveolar bone loss. *J. Dent. Res.* **101**, 216–225 (2022). 34328027 10.1177/00220345211027811

[164] Miyamoto, T. Regulators of osteoclast differentiation and cell-cell fusion. *Keio J. Med.* **60**, 101–105 (2011). 22200633 10.2302/kjm.60.101

[165] Matsubara, T., Yasuda, K., Mizuta, K., Kawaue, H. & Kokabu, S. Tyrosine kinase Src is a regulatory factor of bone homeostasis. *Int. J. Mol. Sci.* **23**, 5508 (2022). 35628319 10.3390/ijms23105508 PMC9146043

[166] Chen, R. et al. Roles of ubiquitin-specific proteases in inflammatory diseases. *Front. Immunol.* **15**, 1258740 (2024). 38322269 10.3389/fimmu.2024.1258740 PMC10844489

[167] Abu-Amer, Y., Ross, F. P., Edwards, J. & Teitelbaum, S. L. Lipopolysaccharide-stimulated osteoclastogenesis is mediated by tumor necrosis factor via its P55 receptor. *J. Clin. Invest.* **100**, 1557–1565 (1997). 9294124 10.1172/JCI119679 PMC508337

[168] Mao, H. Q. et al. STING inhibition alleviates bone resorption in apical periodontitis. *Int. Endod. J.* **57**, 951–965 (2024). 38411951 10.1111/iej.14057

[169] Kleinert, H., Art, J. & Pautz, A. in *Nitric Oxide (Second Edition)* 211–267 (2010).

[170] Piiper, A. & Zeuzem, S. Receptor tyrosine kinases are signaling intermediates of G protein-coupled receptors. *Curr. Pharm. Des.* **10**, 3539–3545 (2004). 15579051 10.2174/1381612043382936

[171] Ambili, R., Santhi, W. S., Janam, P., Nandakumar, K. & Pillai, M. R. Expression of activated transcription factor nuclear factor-kappaB in periodontally diseased tissues. *J. Periodontol.* **76**, 1148–1153 (2005). 16018758 10.1902/jop.2005.76.7.1148

[172] Garcia de Aquino, S. et al. Signaling pathways associated with the expression of inflammatory mediators activated during the course of two models of experimental periodontitis. *Life Sci.* **84**, 745–754 (2009). 19285515 10.1016/j.lfs.2009.03.001

[173] Hayden, M. S. & Ghosh, S. Regulation of NF-kappaB by TNF family cytokines. *Semin Immunol.* **26**, 253–266 (2014). 24958609 10.1016/j.smim.2014.05.004 PMC4156877

[174] Hinz, M. & Scheidereit, C. The IkappaB kinase complex in NF-kappaB regulation and beyond. *EMBO Rep.* **15**, 46–61 (2014). 24375677 10.1002/embr.201337983 PMC4303448

[175] Trares, K., Ackermann, J. & Koch, I. The canonical and non-canonical NF-kappaB pathways and their crosstalk: a comparative study based on Petri nets. *Biosystems* **211**, 104564 (2022). 34688841 10.1016/j.biosystems.2021.104564

[176] Sun, S. C. The non-canonical NF-kappaB pathway in immunity and inflammation. *Nat. Rev. Immunol.* **17**, 545–558 (2017). 28580957 10.1038/nri.2017.52 PMC5753586

[177] Snyder, N. A. & Silva, G. M. Deubiquitinating enzymes (DUBs): regulation, homeostasis, and oxidative stress response. *J. Biol. Chem.* **297**, 101077 (2021). 34391779 10.1016/j.jbc.2021.101077 PMC8424594

[178] Steelman, L. S. et al. Roles of the Raf/MEK/ERK and PI3K/PTEN/Akt/mTOR pathways in controlling growth and sensitivity to therapy-implications for cancer and aging. *Aging* **3**, 192–222 (2011). 21422497 10.18632/aging.100296 PMC3091517

[179] Guo, Q. et al. NF-kappaB in biology and targeted therapy: new insights and translational implications. *Signal Transduct. Target Ther.* **9**, 53 (2024). 38433280 10.1038/s41392-024-01757-9 PMC10910037

[180] Boyce, B. F., Li, J., Yao, Z. & Xing, L. Nuclear factor-kappa B regulation of osteoclastogenesis and osteoblastogenesis. *Endocrinol. Metab.* **38**, 504–521 (2023). 10.3803/EnM.2023.501 PMC10613774 37749800

[181] Makarov, S. S. & NF-kappa, B. in rheumatoid arthritis: a pivotal regulator of inflammation, hyperplasia, and tissue destruction. *Arthritis Res.* **3**, 200–206 (2001). 11438035 10.1186/ar300 PMC128895

[182] Roskoski, R. Jr Janus kinase (JAK) inhibitors in the treatment of inflammatory and neoplastic diseases. *Pharm. Res.* **111**, 784–803 (2016). 10.1016/j.phrs.2016.07.038 27473820

[183] Chen, X. et al. Macrophage M1 polarization mediated via the IL-6/STAT3 pathway contributes to apical periodontitis induced by Porphyromonas gingivalis. *J. Appl. Oral. Sci.* **30**, e20220316 (2022). 36417596 10.1590/1678-7757-2022-0316 PMC9724497

[184] Arce, M. et al. Increased STAT3 activation in periodontitis drives inflammatory bone loss. *J. Dent. Res.* **102**, 1366–1375 (2023). 37697911 10.1177/00220345231192381 PMC10714379

[185] Chaves de Souza, J. A. et al. SOCS3 expression correlates with severity of inflammation, expression of proinflammatory cytokines, and activation of STAT3 and p38 MAPK in LPS-induced inflammation in vivo. *Mediators Inflamm.* **2013**, 650812 (2013). 24078776 10.1155/2013/650812 PMC3775441

[186] Bahar, M. E., Kim, H. J. & Kim, D. R. Targeting the RAS/RAF/MAPK pathway for cancer therapy: from mechanism to clinical studies. *Signal Transduct. Target Ther.* **8**, 455 (2023). 38105263 10.1038/s41392-023-01705-z PMC10725898

[187] Lue, H., Dewor, M., Leng, L., Bucala, R. & Bernhagen, J. Activation of the JNK signalling pathway by macrophage migration inhibitory factor (MIF) and dependence on CXCR4 and CD74. *Cell Signal* **23**, 135–144 (2011). 20807568 10.1016/j.cellsig.2010.08.013 PMC3586206

[188] Kaneko, H. et al. Inhibition of c-Jun N-terminal kinase signaling promotes osteoblastic differentiation of periodontal ligament stem cells and induces regeneration of periodontal tissues. *Arch. Oral. Biol.* **134**, 105323 (2022). 34896864 10.1016/j.archoralbio.2021.105323

[189] Amarasekara, D. S. et al. Regulation of osteoclast differentiation by cytokine networks. *Immune Netw.* **18**, e8 (2018). 29503739 10.4110/in.2018.18.e8 PMC5833125

[190] Perdiguero, E., Ruiz-Bonilla, V., Serrano, A. L. & Munoz-Canoves, P. Genetic deficiency of p38alpha reveals its critical role in myoblast cell cycle exit: the p38alpha-JNK connection. *Cell Cycle* **6**, 1298–1303 (2007). 17534150 10.4161/cc.6.11.4315

[191] Wang, Z. et al. Phosphatase-mediated crosstalk control of ERK and p38 MAPK signaling in corneal epithelial cells. *Investig. Ophthalmol. Vis. Sci.* **47**, 5267–5275 (2006). 17122112 10.1167/iovs.06-0642

[192] Rogers, J. E. et al. A p38 mitogen-activated protein kinase inhibitor arrests active alveolar bone loss in a rat periodontitis model. *J. Periodontol.* **78**, 1992–1998 (2007). 18062121 10.1902/jop.2007.070101

[193] Li, Q. et al. Silencing mitogen-activated protein kinase-activated protein kinase-2 arrests inflammatory bone loss. *J. Pharm. Exp. Ther.* **336**, 633–642 (2011). 10.1124/jpet.110.172395 PMC3061533 21139061

[194] Jiang, L. et al. The proteasome inhibitor bortezomib inhibits inflammatory response of periodontal ligament cells and ameliorates experimental periodontitis in rats. *J. Periodontol.* **88**, 473–483 (2017). 27982724 10.1902/jop.2016.160396

[195] Liu, C. et al. Research on the role and mechanism of the PI3K/Akt/mTOR signalling pathway in osteoporosis. *Front. Endocrinol.* **16**, 1541714 (2025). 10.3389/fendo.2025.1541714 PMC12104071 40421249

[196] Kang, H., Chang, W., Hurley, M., Vignery, A. & Wu, D. Important roles of PI3Kgamma in osteoclastogenesis and bone homeostasis. *Proc. Natl. Acad. Sci. USA* **107**, 12901–12906 (2010). 20616072 10.1073/pnas.1001499107 PMC2919938

[197] Moon, J. B. et al. Akt induces osteoclast differentiation through regulating the GSK3beta/NFATc1 signaling cascade. *J. Immunol.* **188**, 163–169 (2012). 22131333 10.4049/jimmunol.1101254

[198] Wong, B. R. et al. TRANCE, a TNF family member, activates Akt/PKB through a signaling complex involving TRAF6 and c-Src. *Mol. Cell* **4**, 1041–1049 (1999). 10635328 10.1016/s1097-2765(00)80232-4

[199] Lv, H. et al. Kurarinone mitigates LPS-induced inflammatory osteolysis by inhibiting osteoclastogenesis through the reduction of ROS levels and suppression of the PI3K/AKT signaling pathway. *Inflammation*10.1007/s10753-025-02244-1 (2025). 10.1007/s10753-025-02244-1 PMC12596340 39871069

[200] Tang, Y., Pan, J., Guo, H. & Li, Q. Identification of an active fraction of Kangfuxin in the treatment of periodontitis in a rat model. *Folia Histochem Cytobiol.* **62**, 133–144 (2024). 39329499 10.5603/fhc.101230

[201] Wang, W. et al. Capsaicin attenuates Porphyromonas gingivalis-suppressed osteogenesis of periodontal ligament stem cells via regulating mitochondrial function and activating PI3K/AKT/mTOR pathway. *J. Periodontal Res.* **59**, 798–811 (2024). 38699845 10.1111/jre.13252

[202] Yu, C. et al. Garlic-derived exosome-like nanovesicles: a promising natural nanotherapy for periodontitis via PHGDH/PI3K/AKT-mediated metabolic and inflammatory regulation. *Int. J. Nanomed.* **20**, 5551–5572 (2025). 10.2147/IJN.S510417 PMC12050027 40321804

[203] Liu, J. et al. Wnt/beta-catenin signalling: function, biological mechanisms, and therapeutic opportunities. *Signal Transduct. Target Ther.* **7**, 3 (2022). 34980884 10.1038/s41392-021-00762-6 PMC8724284

[204] Zhan, T., Rindtorff, N. & Boutros, M. Wnt signaling in cancer. *Oncogene* **36**, 1461–1473 (2017). 27617575 10.1038/onc.2016.304 PMC5357762

[205] Bao, J., Yang, Y., Xia, M., Sun, W. & Chen, L. Wnt signaling: an attractive target for periodontitis treatment. *Biomed. Pharmacother.* **133**, 110935 (2021). 33227711 10.1016/j.biopha.2020.110935

[206] Duan, P. & Bonewald, L. F. The role of the wnt/beta-catenin signaling pathway in formation and maintenance of bone and teeth. *Int. J. Biochem. Cell Biol.* **77**, 23–29 (2016). 27210503 10.1016/j.biocel.2016.05.015 PMC4958569

[207] Tu, X. et al. Osteocytes mediate the anabolic actions of canonical Wnt/beta-catenin signaling in bone. *Proc. Natl. Acad. Sci. USA* **112**, E478–486 (2015). 25605937 10.1073/pnas.1409857112 PMC4321271

[208] Zhou, M. & Graves, D. T. Impact of the host response and osteoblast lineage cells on periodontal disease. *Front. Immunol.* **13**, 998244 (2022). 36304447 10.3389/fimmu.2022.998244 PMC9592920

[209] Nishi, H., Demir, E. & Panchenko, A. R. Crosstalk between signaling pathways provided by single and multiple protein phosphorylation sites. *J. Mol. Biol.* **427**, 511–520 (2015). 25451034 10.1016/j.jmb.2014.11.001 PMC4297578

[210] Chu, X. et al. The role of senescence in experimental periodontitis at the causal level: an in vivo study. *Cells* **14**, 226 (2025). 39937017 10.3390/cells14030226 PMC11817363

[211] Xu, Y., Li, N., Xiang, R. & Sun, P. Emerging roles of the p38 MAPK and PI3K/AKT/mTOR pathways in oncogene-induced senescence. *Trends Biochem. Sci.* **39**, 268–276 (2014). 24818748 10.1016/j.tibs.2014.04.004 PMC4358807

[212] Li, H. et al. Transcriptional factor HBP1 targets P16(INK4A), upregulating its expression and consequently is involved in Ras-induced premature senescence. *Oncogene* **29**, 5083–5094 (2010). 20581871 10.1038/onc.2010.252

[213] Borodkina, A., Shatrova, A., Abushik, P., Nikolsky, N. & Burova, E. Interaction between ROS dependent DNA damage, mitochondria and p38 MAPK underlies senescence of human adult stem cells. *Aging* **6**, 481–495 (2014). 24934860 10.18632/aging.100673 PMC4100810

[214] Freund, A., Patil, C. K. & Campisi, J. p38MAPK is a novel DNA damage response-independent regulator of the senescence-associated secretory phenotype. *EMBO J.* **30**, 1536–1548 (2011). 21399611 10.1038/emboj.2011.69 PMC3102277

[215] Yin, M., Zheng, X. & Shi, L. Targeting p38 MAPK: a potential bridge between ER stress and age-related bone loss. *Cell Signal* **127**, 111549 (2025). 39638139 10.1016/j.cellsig.2024.111549

[216] Oliveira, G. E. et al. Exploring the impact of biological agents on protecting against experimental periodontitis: a systematic review of animal-based studies. *Biomed. Res. Int.* **2024**, 1716735 (2024). 39654845 10.1155/bmri/1716735 PMC11628168

[217] Pavanelli, A. L. R. et al. Pharmacological therapies for the management of inflammatory bone resorption in periodontal disease: a review of preclinical studies. *Biomed. Res. Int.* **2022**, 5832009 (2022). 35547360 10.1155/2022/5832009 PMC9085331

[218] Boyce, B. F. et al. Future anti-catabolic therapeutic targets in bone disease. *Ann. N. Y. Acad. Sci.* **1068**, 447–457 (2006). 16831942 10.1196/annals.1346.042

[219] Yang, B., Pang, X., Li, Z., Chen, Z. & Wang, Y. Immunomodulation in the treatment of periodontitis: progress and perspectives. *Front. Immunol.* **12**, 781378 (2021). 34868054 10.3389/fimmu.2021.781378 PMC8640126

[220] Wu, Y. et al. Bone targeted nano-drug and nano-delivery. *Bone Res.* **12**, 51 (2024). 39231955 10.1038/s41413-024-00356-2 PMC11375042

[221] Stapleton, M. et al. Development of bone targeting drugs. *Int. J. Mol. Sci.* **18**, 1345 (2017). 28644392 10.3390/ijms18071345 PMC5535838

[222] de Molon, R. S. et al. Evaluation of the host response in various models of induced periodontal disease in mice. *J. Periodontol.* **85**, 465–477 (2014). 23805811 10.1902/jop.2013.130225

[223] de Molon, R. S. et al. Long-term evaluation of oral gavage with periodontopathogens or ligature induction of experimental periodontal disease in mice. *Clin. Oral. Investig.* **20**, 1203–1216 (2016). 26411857 10.1007/s00784-015-1607-0

[224] de Molon, R. S., Park, C. H., Jin, Q., Sugai, J. & Cirelli, J. A. Characterization of ligature-induced experimental periodontitis. *Microsc Res. Tech.* **81**, 1412–1421 (2018). 30351474 10.1002/jemt.23101

[225] de Molon, R. S., de Avila, E. D. & Cirelli, J. A. Host responses induced by different animal models of periodontal disease: a literature review. *J. Investig. Clin. Dent.* **4**, 211–218 (2013). 23188588 10.1111/jicd.12018

[226] Koide, M., Kinugawa, S., Takahashi, N. & Udagawa, N. Osteoclastic bone resorption induced by innate immune responses. *Periodontol 2000* **54**, 235–246 (2010). 20712643 10.1111/j.1600-0757.2010.00355.x

[227] Silva, B. R. et al. Establishing a dual murine model to explore the interactions between diabetes and periodontitis in mice. *Int. J. Mol. Sci.* **26**, 5611 (2025). 40565075 10.3390/ijms26125611 PMC12192739

[228] Zhen, L., Fan, D. S., Zhang, Y., Cao, X. M. & Wang, L. M. Resveratrol ameliorates experimental periodontitis in diabetic mice through negative regulation of TLR4 signaling. *Acta Pharm. Sin.* **36**, 221–228 (2015). 10.1038/aps.2014.131 PMC4326790 25530164

[229] Xiao, C. J., Yu, X. J., Xie, J. L., Liu, S. & Li, S. Protective effect and related mechanisms of curcumin in rat experimental periodontitis. *Head. Face Med.* **14**, 12 (2018). 30115081 10.1186/s13005-018-0169-1 PMC6097422

[230] Elburki, M. S. et al. A chemically modified curcumin (CMC 2.24) inhibits nuclear factor kappaB activation and inflammatory bone loss in murine models of LPS-induced experimental periodontitis and diabetes-associated natural periodontitis. *Inflammation* **40**, 1436–1449 (2017). 28534138 10.1007/s10753-017-0587-4

[231] Wong, M. et al. TNFalpha blockade in human diseases: mechanisms and future directions. *Clin. Immunol.* **126**, 121–136 (2008). 17916444 10.1016/j.clim.2007.08.013 PMC2291518

[232] Dinarello, C. A. Overview of the IL-1 family in innate inflammation and acquired immunity. *Immunol. Rev.* **281**, 8–27 (2018). 29247995 10.1111/imr.12621 PMC5756628

[233] McIntyre, K. W. et al. A highly selective inhibitor of I kappa B kinase, BMS-345541, blocks both joint inflammation and destruction in collagen-induced arthritis in mice. *Arthritis Rheum.* **48**, 2652–2659 (2003). 13130486 10.1002/art.11131

[234] Gillooly, K. M. et al. Periodic, partial inhibition of IkappaB Kinase beta-mediated signaling yields therapeutic benefit in preclinical models of rheumatoid arthritis. *J. Pharm. Exp. Ther.* **331**, 349–360 (2009). 10.1124/jpet.109.156018 19652024

[235] Huang, Y. K. IL-8 as a potential therapeutic target for periodontitis and its inhibition by caffeic acid phenethyl ester in vitro. *Int. J. Mol. Sci.* **22**, 3641 (2021). 33807391 10.3390/ijms22073641 PMC8037988

[236] Aoki, T. et al. Inhibition of non-canonical NF-kappaB signaling suppresses periodontal inflammation and bone loss. *Front. Immunol.* **14**, 1179007 (2023). 37143646 10.3389/fimmu.2023.1179007 PMC10151688

[237] Wang, Y. et al. AZD8835 inhibits osteoclastogenesis and periodontitis-induced alveolar bone loss in rats. *J. Cell Physiol.* **234**, 10432–10444 (2019). 30652303 10.1002/jcp.27711

[238] Jimi, E. et al. Selective inhibition of NF-kappa B blocks osteoclastogenesis and prevents inflammatory bone destruction in vivo. *Nat. Med.* **10**, 617–624 (2004). 15156202 10.1038/nm1054

[239] Zhang, Z. et al. NAT10 regulates the LPS-induced inflammatory response via the NOX2-ROS-NF-kappaB pathway in macrophages. *Biochim. Biophys. Acta Mol. Cell Res.* **1870**, 119521 (2023). 37307924 10.1016/j.bbamcr.2023.119521

[240] Zhu, Y., Qiao, S., Pang, Y., Wang, H. & Zhou, Y. Deferoxamine treatment effectively prevents periodontitis progression by reducing inflammation and osteoclastogenesis in experimental periodontitis rats. *J. Inflamm. Res.* **17**, 9637–9650 (2024). 39618936 10.2147/JIR.S490823 PMC11606167

[241] Cafferata, E. A. et al. Interleukin-35 inhibits alveolar bone resorption by modulating the Th17/Treg imbalance during periodontitis. *J. Clin. Periodontol.* **47**, 676–688 (2020). 32160331 10.1111/jcpe.13282

[242] Li, L. et al. IL-37 alleviates alveolar bone resorption and inflammatory response through the NF-kappaB/NLRP3 signaling pathway in male mice with periodontitis. *Arch. Oral. Biol.* **147**, 105629 (2023). 36680836 10.1016/j.archoralbio.2023.105629

[243] Wang, B. et al. TPCA-1 negatively regulates inflammation mediated by NF-kappaB pathway in mouse chronic periodontitis model. *Mol. Oral. Microbiol.* **36**, 192–201 (2021). 33768683 10.1111/omi.12335

[244] Wang, J. et al. Inhibition of nuclear factor kappa B inducing kinase suppresses inflammatory responses and the symptoms of chronic periodontitis in a mouse model. *Int. J. Biochem. Cell Biol.* **139**, 106052 (2021). 34364989 10.1016/j.biocel.2021.106052

[245] Girisa, S. et al. From simple mouth cavities to complex oral mucosal disorders-curcuminoids as a promising therapeutic approach. *ACS Pharm. Transl. Sci.* **4**, 647–665 (2021). 10.1021/acsptsci.1c00017 PMC8033761 33860191

[246] Minagawa, T. et al. Resveratrol suppresses the inflammatory responses of human gingival epithelial cells in a SIRT1 independent manner. *J. Periodontal Res.* **50**, 586–593 (2015). 25312218 10.1111/jre.12238

[247] Tan, Y., Feng, J., Xiao, Y. & Bao, C. Grafting resveratrol onto mesoporous silica nanoparticles towards efficient sustainable immunoregulation and insulin resistance alleviation for diabetic periodontitis therapy. *J. Mater. Chem. B* **10**, 4840–4855 (2022). 35678150 10.1039/d2tb00484d

[248] Huangfu, H. et al. Facile engineering of resveratrol nanoparticles loaded with 20(S)-protopanaxadiol for the treatment of periodontitis by regulating the macrophage phenotype. *Nanoscale* **15**, 7894–7908 (2023). 37060139 10.1039/d2nr06452a

[249] Zhu, Y. et al. A Chitosan-based hydrogel to modulate immune cells and promote periodontitis healing in the high-fat diet-induced periodontitis rat model. *Acta Biomater.* **200**, 452–463 (2025). 40379118 10.1016/j.actbio.2025.05.034

[250] de Molon, R. S. Therapeutic potential of tanshinones in osteolytic diseases: from molecular and cellular pathways to preclinical models. *Dent. J.* **9**, 309 (2025). 10.3390/dj13070309 PMC12293886 40710154

[251] Pavanelli, A. L. R. et al. Anti-inflammatory and antiresorptive activities of tanshinone-IIA mitigate alveolar bone destruction in mice with experimental periodontitis. *J. Periodontol.*10.1002/JPER.24-0618 (2025). 10.1002/JPER.24-0618 40663013

[252] Cafferata, E. A. et al. Boldine inhibits the alveolar bone resorption during ligature-induced periodontitis by modulating the Th17/Treg imbalance. *J. Periodontol.* **92**, 123–136 (2021). 32490537 10.1002/JPER.20-0055

[253] Liu, H. et al. Hesperetin suppresses RANKL-induced osteoclastogenesis and ameliorates lipopolysaccharide-induced bone loss. *J. Cell Physiol.* **234**, 11009–11022 (2019). 30548260 10.1002/jcp.27924

[254] Goncalves, V. P. et al. Systemic dietary hesperidin modulation of osteoclastogenesis, bone homeostasis and periodontal disease in mice. *Int. J. Mol. Sci* **23**, 7100 (2022). 35806105 10.3390/ijms23137100 PMC9266620

[255] Fu, J. et al. Lactobacillus rhamnosus inhibits osteoclast differentiation by suppressing the TLR2/NF-kappaB pathway. *Oral. Dis.* **30**, 2373–2386 (2024). 37602540 10.1111/odi.14712

[256] Marotte, H. & Miossec, P. Prevention of bone mineral density loss in patients with rheumatoid arthritis treated with anti-TNFalpha therapy. *Biologics* **2**, 663–669 (2008). 19707447 10.2147/btt.s2338 PMC2727908

[257] Goncalves, D. C. et al. Infliximab attenuates inflammatory osteolysis in a model of periodontitis in Wistar rats. *Exp. Biol. Med.* **239**, 442–453 (2014). 10.1177/1535370213520114 24586097

[258] Dinarello, C. A., Simon, A. & van der Meer, J. W. Treating inflammation by blocking interleukin-1 in a broad spectrum of diseases. *Nat. Rev. Drug Discov.* **11**, 633–652 (2012). 22850787 10.1038/nrd3800 PMC3644509

[259] Wang, F. et al. IL-1beta receptor antagonist (IL-1Ra) combined with autophagy inducer (TAT-Beclin1) is an effective alternative for attenuating extracellular matrix degradation in rat and human osteoarthritis chondrocytes. *Arthritis Res. Ther.* **21**, 171 (2019). 31291980 10.1186/s13075-019-1952-5 PMC6617669

[260] Lee, S. Y. et al. IL-1 receptor antagonist (IL-1Ra)-Fc ameliorate autoimmune arthritis by regulation of the Th17 cells/Treg balance and arthrogenic cytokine activation. *Immunol. Lett.* **172**, 56–66 (2016). 26903194 10.1016/j.imlet.2016.02.011

[261] Liu, Y. et al. Treatment of periodontal inflammation in diabetic rats with IL-1ra thermosensitive hydrogel. *Int. J. Mol. Sci.* **23**, 13939 (2022). 36430410 10.3390/ijms232213939 PMC9693501

[262] Hashimoto, T. et al. Tocilizumab suppresses NF-kappa B activation via toll-like receptor 9 signaling by reducing cell-free DNA in rheumatoid arthritis. *Clin. Exp. Immunol.* **213**, 209–220 (2023). 37279559 10.1093/cei/uxad064 PMC10361738

[263] Apolinario Vieira, G. H. et al. Specific inhibition of IL-6 receptor attenuates inflammatory bone loss in experimental periodontitis. *J. Periodontol.* **92**, 1460–1469 (2021). 33492708 10.1002/JPER.20-0455

[264] Koide, M. et al. Osteoprotegerin-deficient male mice as a model for severe alveolar bone loss: comparison with RANKL-overexpressing transgenic male mice. *Endocrinology* **154**, 773–782 (2013). 23291450 10.1210/en.2012-1928

[265] Cummings, S. R. et al. Denosumab for prevention of fractures in postmenopausal women with osteoporosis. *N. Engl. J. Med.* **361**, 756–765 (2009). 19671655 10.1056/NEJMoa0809493

[266] Yuan, H., Gupte, R., Zelkha, S. & Amar, S. Receptor activator of nuclear factor kappa B ligand antagonists inhibit tissue inflammation and bone loss in experimental periodontitis. *J. Clin. Periodontol.* **38**, 1029–1036 (2011). 22092474 10.1111/j.1600-051X.2011.01780.x

[267] de Molon, R. S. et al. Spontaneous osteonecrosis of the jaws in the maxilla of mice on antiresorptive treatment: a novel ONJ mouse model. *Bone* **68**, 11–19 (2014). 25093262 10.1016/j.bone.2014.07.027 PMC4476062

[268] de Molon, R. S. et al. OPG-Fc but not zoledronic acid discontinuation reverses osteonecrosis of the jaws (ONJ) in mice. *J. Bone Miner. Res* **30**, 1627–1640 (2015). 25727550 10.1002/jbmr.2490 PMC4995600

[269] Soundia, A. et al. Osteonecrosis of the jaws (ONJ) in mice after extraction of teeth with periradicular disease. *Bone* **90**, 133–141 (2016). 27327410 10.1016/j.bone.2016.06.011 PMC5471352

[270] de Molon, R. S. et al. Rheumatoid arthritis exacerbates the severity of osteonecrosis of the jaws (ONJ) in mice. a randomized, prospective, controlled animal study. *J. Bone Miner. Res.* **31**, 1596–1607 (2016). 26950411 10.1002/jbmr.2827 PMC4970902

[271] Nakamura, M. et al. Osteoprotegerin regulates bone formation through a coupling mechanism with bone resorption. *Endocrinology* **144**, 5441–5449 (2003). 14500574 10.1210/en.2003-0717

[272] Jin, Q. et al. RANKL inhibition through osteoprotegerin blocks bone loss in experimental periodontitis. *J. Periodontol.* **78**, 1300–1308 (2007). 17608585 10.1902/jop.2007.070073 PMC2583091

[273] Hamar, A. et al. Effects of one-year tofacitinib therapy on bone metabolism in rheumatoid arthritis. *Osteoporos. Int.* **32**, 1621–1629 (2021). 33559714 10.1007/s00198-021-05871-0 PMC8376736

[274] Kobayashi, T., Ito, S., Murasawa, A., Ishikawa, H. & Yoshie, H. Effects of tofacitinib on the clinical features of periodontitis in patients with rheumatoid arthritis: two case reports. *BMC Rheumatol.* **3**, 13 (2019). 31020271 10.1186/s41927-019-0062-y PMC6474052

[275] Yarilina, A., Xu, K., Chan, C. & Ivashkiv, L. B. Regulation of inflammatory responses in tumor necrosis factor-activated and rheumatoid arthritis synovial macrophages by JAK inhibitors. *Arthritis Rheum.* **64**, 3856–3866 (2012). 22941906 10.1002/art.37691 PMC3510320

[276] Li, C. H. et al. Stattic inhibits RANKL-mediated osteoclastogenesis by suppressing activation of STAT3 and NF-kappaB pathways. *Int. Immunopharmacol.* **58**, 136–144 (2018). 29587202 10.1016/j.intimp.2018.03.021

[277] Li, Q., Valerio, M. S. & Kirkwood, K. L. MAPK usage in periodontal disease progression. *J. Signal Transduct.* **2012**, 308943 (2012). 22315682 10.1155/2012/308943 PMC3270463

[278] Gu, L., Ke, Y., Gan, J. & Li, X. Berberine suppresses bone loss and inflammation in ligature-induced periodontitis through promotion of the G protein-coupled estrogen receptor-mediated inactivation of the p38MAPK/NF-kappaB pathway. *Arch. Oral. Biol.* **122**, 104992 (2021). 33338754 10.1016/j.archoralbio.2020.104992

[279] Wang, C. et al. Role of berberine thermosensitive hydrogel in periodontitis via PI3K/AKT pathway in vitro. *Int. J. Mol. Sci.* **24**, 6364 (2023). 37047340 10.3390/ijms24076364 PMC10094121

[280] Zhao, B. et al. Eupatilin suppresses osteoclastogenesis and periodontal bone loss by inhibiting the MAPKs/Siglec-15 pathway. *Int. Immunopharmacol.* **139**, 112720 (2024). 39047450 10.1016/j.intimp.2024.112720

[281] Chen, Y. et al. The effect of the Litcubanine A on the treatment of murine experimental periodontitis by inhibiting monocyte-macrophage chemotaxis and osteoclast differentiation. *J. Periodontal Res.* **58**, 948–958 (2023). 37409514 10.1111/jre.13154

[282] Yang, J. et al. The effect of the root bark of Lycium chinense (Lycii Radicis Cortex) on experimental periodontitis and alveolar bone loss in Sprague-Dawley rats. *Antioxidants* **13**, 1332 (2024). 39594474 10.3390/antiox13111332 PMC11590933

[283] Kim, J. A. et al. Napyradiomycin B4 suppresses RANKL-induced osteoclastogenesis and prevents alveolar bone destruction in experimental periodontitis. *ACS Pharm. Transl. Sci.* **7**, 1023–1031 (2024). 10.1021/acsptsci.3c00315 PMC11019734 38633588

[284] Liu, C. L. et al. Ugonin L inhibits osteoclast formation and promotes osteoclast apoptosis by inhibiting the MAPK and NF-kappaB pathways. *Biomed. Pharmacother.* **166**, 115392 (2023). 37651802 10.1016/j.biopha.2023.115392

[285] Shuai, F. et al. A nucleoside-based supramolecular hydrogel integrating localized self-delivery and immunomodulation for periodontitis treatment. *Biomaterials* **316**, 123024 (2025). 39705922 10.1016/j.biomaterials.2024.123024

[286] Sun, M. et al. Ginsenoside Rb3 inhibits osteoclastogenesis via ERK/NF-kappaB signaling pathway in vitro and in vivo. *Oral. Dis.* **29**, 3460–3471 (2023). 35976062 10.1111/odi.14352

[287] Wang, Z., Zhan, C., Zeng, F. & Wu, S. A biopolymer-based and inflammation-responsive nanodrug for rheumatoid arthritis treatment via inhibiting JAK-STAT and JNK signalling pathways. *Nanoscale* **12**, 23013–23027 (2020). 33191426 10.1039/d0nr05551d

[288] Han, Z. et al. c-Jun N-terminal kinase is required for metalloproteinase expression and joint destruction in inflammatory arthritis. *J. Clin. Invest.* **108**, 73–81 (2001). 11435459 10.1172/JCI12466 PMC209341

[289] Sun, Y. et al. Intermittent hyperglycaemia induces macrophage dysfunction by extracellular regulated protein kinase-dependent PKM2 translocation in periodontitis. *Cell Prolif.* **57**, e13651 (2024). 38790140 10.1111/cpr.13651 PMC11471441

[290] Ihn, H. J. et al. PF-3845, a fatty acid amide hydrolase inhibitor, directly suppresses osteoclastogenesis through ERK and NF-kappaB pathways in vitro and alveolar bone loss in vivo. *Int. J. Mol. Sci.* **22**, 1915 (2021). 33671948 10.3390/ijms22041915 PMC7919013

[291] Goldstein, D. M. & Gabriel, T. Pathway to the clinic: inhibition of P38 MAP kinase. A review of ten chemotypes selected for development. *Curr. Top. Med. Chem.* **5**, 1017–1029 (2005). 16178744 10.2174/1568026054985939

[292] Pargellis, C. & Regan, J. Inhibitors of p38 mitogen-activated protein kinase for the treatment of rheumatoid arthritis. *Curr. Opin. Investig. Drugs* **4**, 566–571 (2003). 12833650

[293] Patil, C. S. & Kirkwood, K. L. p38 MAPK signaling in oral-related diseases. *J. Dent. Res.* **86**, 812–825 (2007). 17720848 10.1177/154405910708600903

[294] Cao, J. et al. Kaempferol combats the osteogenic differentiation damage of periodontal ligament stem cells in periodontitis via regulating EphrinB2-mediated PI3K/Akt and P38 pathways. *Phytomedicine* **141**, 156733 (2025). 40220409 10.1016/j.phymed.2025.156733

[295] Mu, H. et al. Inhibition of fibulin-3 ameliorates periodontal inflammation through reducing M1 macrophage polarization via EGFR/PI3K/AKT pathway. *J. Periodontol.* **96**, 440–454 (2025). 39692480 10.1002/JPER.24-0405

[296] Socransky, S. S., Haffajee, A. D., Goodson, J. M. & Lindhe, J. New concepts of destructive periodontal disease. *J. Clin. Periodontol.* **11**, 21–32 (1984). 6582072 10.1111/j.1600-051x.1984.tb01305.x

[297] Goodson, J. M., Tanner, A. C., Haffajee, A. D., Sornberger, G. C. & Socransky, S. S. Patterns of progression and regression of advanced destructive periodontal disease. *J. Clin. Periodontol.* **9**, 472–481 (1982). 6960023 10.1111/j.1600-051x.1982.tb02108.x

[298] Graves, D. T., Fine, D., Teng, Y. T., Van Dyke, T. E. & Hajishengallis, G. The use of rodent models to investigate host-bacteria interactions related to periodontal diseases. *J. Clin. Periodontol.* **35**, 89–105 (2008). 18199146 10.1111/j.1600-051X.2007.01172.x PMC2649707

[299] Kinney, J. S. et al. Saliva/pathogen biomarker signatures and periodontal disease progression. *J. Dent. Res.* **90**, 752–758 (2011). 21406610 10.1177/0022034511399908 PMC3144122

[300] Ramseier, C. A. et al. Identification of pathogen and host-response markers correlated with periodontal disease. *J. Periodontol.* **80**, 436–446 (2009). 19254128 10.1902/jop.2009.080480 PMC5695217

[301] Yilmaz, B. et al. Dual-drug carboxymethyl chitosan hydrogel: development, characterization, and in vitro evaluation for periodontal therapy. *Carbohydr. Polym.* **363**, 123726 (2025). 40441835 10.1016/j.carbpol.2025.123726

[302] Yin, B. et al. Smart injectable hydrogels for periodontal regeneration: Recent advancements in biomaterials and biofabrication strategies. *Mater. Today Bio* **32**, 101855 (2025). 40487163 10.1016/j.mtbio.2025.101855 PMC12145717

[303] Nakajima, M. et al. Advances in local drug delivery for periodontal treatment: present strategies and future directions. *Biomolecules* **15**, 903 (2025). 40563543 10.3390/biom15060903 PMC12191426

[304] Hashim, N. T. et al. New insights in natural bioactive compounds for periodontal disease: advanced molecular mechanisms and therapeutic potential. *Molecules* **30**, 807 (2025). 40005119 10.3390/molecules30040807 PMC11858609

[305] Lopez-Valverde, N. et al. Antioxidant, anti-inflammatory and antimicrobial activity of natural products in periodontal disease: a comprehensive review. *Front. Bioeng. Biotechnol.* **11**, 1226907 (2023). 37600299 10.3389/fbioe.2023.1226907 PMC10435350

[306] Anwar, M. A. et al. Herbal remedies for oral and dental health: a comprehensive review of their multifaceted mechanisms including antimicrobial, anti-inflammatory, and antioxidant pathways. *Inflammopharmacology* **33**, 1085–1160 (2025). 39907951 10.1007/s10787-024-01631-8 PMC11914039

[307] Hashim, N. T. et al. Natural bioactive compounds in the management of periodontal diseases: a comprehensive review. *Molecules* **29**, 3044 (2024). 38998994 10.3390/molecules29133044 PMC11242977

